# Design, Microwave-Assisted Synthesis and In Silico Prediction Study of Novel Isoxazole Linked Pyranopyrimidinone Conjugates as New Targets for Searching Potential Anti-SARS-CoV-2 Agents

**DOI:** 10.3390/molecules26206103

**Published:** 2021-10-10

**Authors:** Faisal K. Algethami, Maher Cherif, Salma Jlizi, Naoufel Ben Hamadi, Anis Romdhane, Mohamed R. Elamin, Mashael A. Alghamdi, Hichem Ben Jannet

**Affiliations:** 1 Department of Chemistry, College of Science, Imam Mohammad Ibn Saud Islamic University (IMSIU), Riyadh 11432, Saudi Arabia; bh_naoufel@yahoo.fr (N.B.H.); mohamedrahmt99@gmail.com (M.R.E.); mabalghamdi@imamu.edu.sa (M.A.A.); 2 Laboratory of Heterocyclic Chemistry, Natural Products and Reactivity (LR11ES39), Medicinal Chemistry and Natural Products Team, Faculty of Science of Monastir, University of Monastir, Avenue of Environment, Monastir 5019, Tunisia; cherifmhr13@gmail.com (M.C.); salma.jlizi@gmail.com (S.J.); anis_romdhane@yahoo.fr (A.R.)

**Keywords:** pyranopyrimidinone, isoxazole, copper catalyst, click chemistry, microwave irradiation, in silicomolecular docking, SARS-CoV-2 M^pro^ inhibitors

## Abstract

A series of novel naphthopyrano[2,3-*d*]pyrimidin-11(12*H*)-one containing isoxazole nucleus **4** was synthesized under microwave irradiation and classical conditions in moderate to excellent yields upon 1,3-dipolar cycloaddition reaction using various arylnitrile oxides under copper(I) catalyst. A one-pot, three-component reaction, *N*-propargylation and Dimroth rearrangement were used as the key steps for the preparation of the dipolarophiles**3**. The structures of the synthesized compounds were established by ^1^H NMR, ^13^C NMR and HRMS-ES means. The present study aims to also predict the theoretical assembly of the COVID-19 protease (SARS-CoV-2 M^pro^) and to discover in advance whether this protein can be targeted by the compounds **4a–1** and thus be synthesized. The docking scores of these compounds were compared to those of the co-crystallized native ligand inhibitor (N3) which was used as a reference standard. The results showed that all the synthesized compounds (**4a–l**) gave interesting binding scores compared to those of N3 inhibitor. It was found that compounds **4a**, **4e** and **4i** achieved greatly similar binding scores and modes of interaction than N3, indicating promising affinity towards SARS-CoV-2 M^pro^. On the other hand, the derivatives **4k**, **4h** and **4j** showed binding energy scores (−8.9, −8.5 and −8.4 kcal/mol, respectively) higher than the M^pro^ N3 inhibitor (−7.0 kcal/mol), revealing, in their turn, a strong interaction with the target protease, although their interactions were not entirely comparable to that of the reference N3.

## 1. Introduction

Coronaviruses (CoVs) tend to have a high zoonotic potential and according to the World Health Organization (WHO), these viral diseases have emerged as a serious health issue to the world [1]. Consequently, the WHO declared a state of global health emergency to coordinate scientific and medical efforts to rapidly develop a cure for patients [2]. The discovery of an antiviral drug is of immense importance in the current spread of the rapidly modifiable SARS-CoV-2. The aim of the present study was to develop an antiviral drug against the novel COVID-19 virus [3]. One of the ways that several research teams are thinking is to highlight promising molecules and drug compounds by making a virtual screening via molecular docking of drugs approved by the FDA, certain natural substances, and synthetic heterocyclic compounds such as pyranopyrimidinones linked to isoxazoles for probable therapeutic outcome. In this context, antiviral compounds have seriously attracted the attention of organic medicinal chemists, who have thought of chemically modifying them in different ways in order to achieve much more active structural analogues.

**Figure 1:**
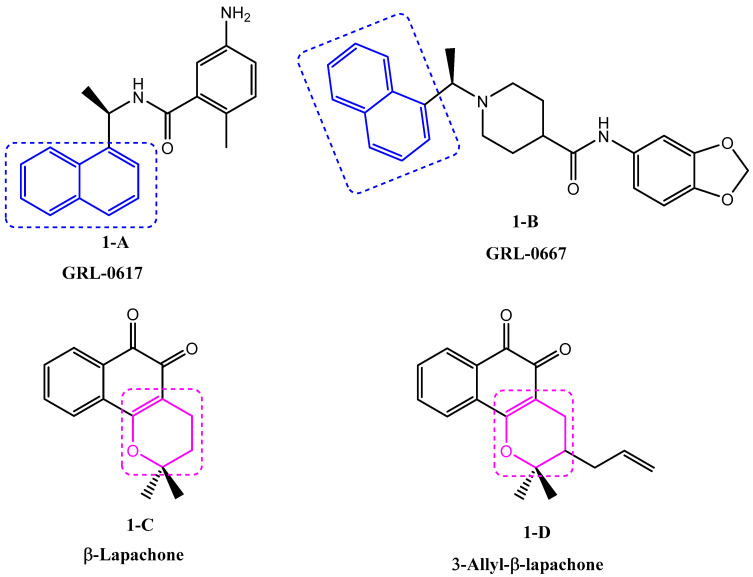
Previously reported antiviral naphthalene and conjugated pyran derivatives.

Naphthalenes have, for a long time, attracted the attention of organic chemists and biologists due to their wide spectrum of biological properties, such as antimicrobial, cytotoxic [4], cardiovascular [5], anti-inflammatory [6], and antiviral (Figure 1, 1-A [7], 1-B [8]).

On the other hand, heterocyclic scaffolds play a central role in drug discovery and development, thus constituting the key structural component of a majority of biologically active moieties. Conjugated pyranes are heterocyclic compounds arousing great interest due to their multiple biological activities, such as antimicrobial [9], anti-tumor [10], influenza inhibition [11] and antiviral (Figure 1, 1-C, 1-D) [12].

**Figure 2:**
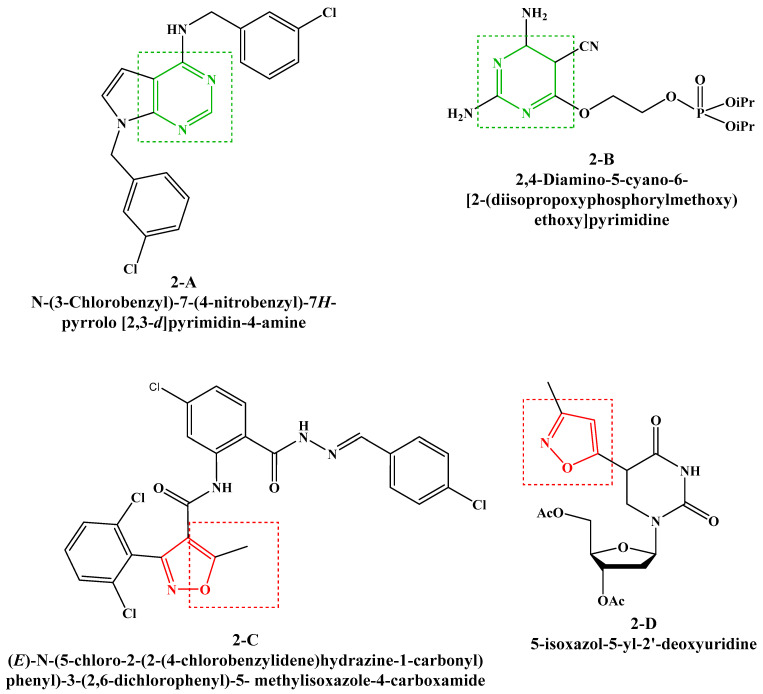
Previously reported antiviral compounds bearing pyrimidine and isoxazole rings.

On the other hand, pyrimidines are among the most important nitrogenous heterocyclic classes in medicinal chemistry due to their various extensive biological and therapeutic activities [13]. In this context, the condensed derivatives of pyrimidines are very attractive targets because of their various pharmacological effects, such as anti-tubercular [14], antitumor [15], antityrosinase [16], antiproliferative [17], anti-inflammatory [18] and antiviral (Figure 2, 2-A [19], 2-B [20]).

Furthermore, isoxazole derivatives possess various biological activities such as anti-inflammatory [21], antileishmanial [22], trypanocidal [23], antimicrobial [24,25] and antiviral (Figure 2, 2-C [26], 2-D [27]).

**Figure 3:**
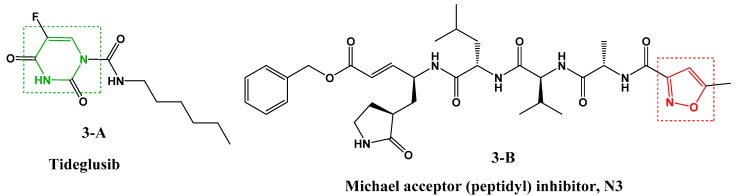
Structure of SARS-CoV-2 M^pro^ inhibitors.

In addition, numerous target-based virtual screenings were performed to discover promising protease inhibitors against SARS-CoV-2. In this context, a recent study showed that high throughput virtual screening of more than 10,000 molecules, based on the SARS-CoV-2 M^pro^ structure, resulted in the identification of six small inhibitors molecules [28], one of which is a pyrimidine derivative (Figure 3, 3-A [28]) and the other contains the isoxazole nucleus (Figure 3, 3-B [28]).

**Figure 4:**
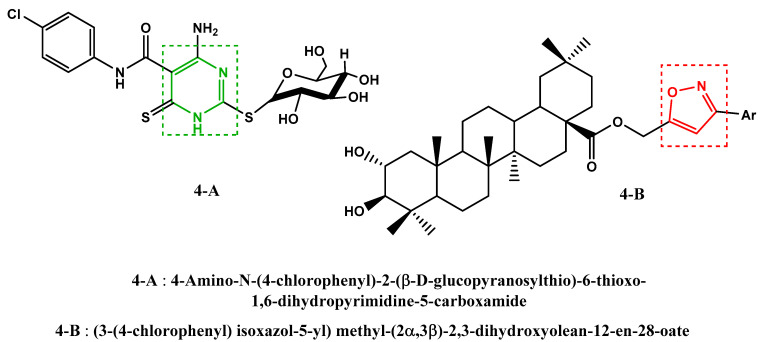
Previously reported anti-SARS-CoV-2 molecules.

Furthermore, recent work has shown that certain compounds, containing in their structures the pyrimidine (Figure 4, 4-A [29]) and isoxazole (Figure 4, 4-B [30]) units, have shown an anti-SARS-CoV-2 activity.

**Scheme 1:**
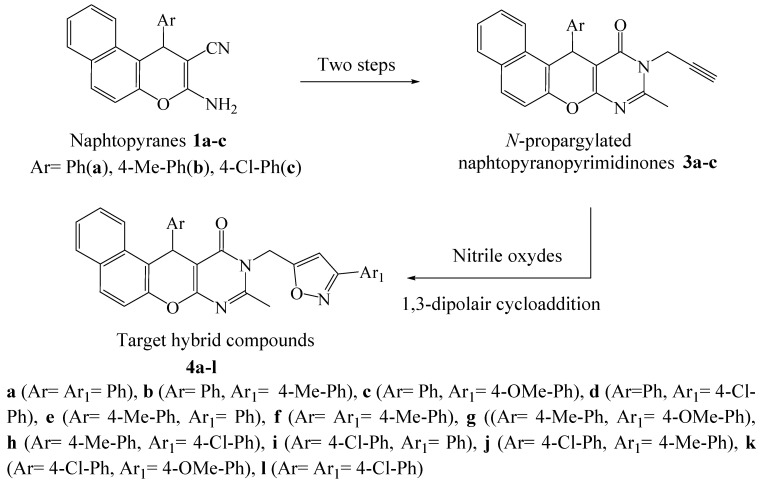
The design of target compounds **4a–l**.

In addition, the junction of the pyrimidine ring with different heterocyclic fragments in the same molecule could lead to a new category of hybrid molecules with pronounced biological power. In this context, and in the continuity of looking for new bioactive pyrimidine compounds [31,32,33], we report herein the synthesis of a new series of hybrid compounds **4**, in three steps (Scheme 1), under conventional heating conditions and by microwave irradiation. We have chosen to integrate into the same molecule the naphthalene, pyran, pyrimidine and isoxazole fragments assigned as antiviral and anti-SARS-CoV-2 agents, as indicated above, with the aim to perform their virtual screening, using molecular docking studies, hoping to find out promising protease inhibitors against COVID-19.

## 2. Results and Discussion

### 2.1. Chemistry

Our key starting materials, 2-amino-3-cyanonaphthopyranes **1a**–**c**, were synthesized by adopting the method of Zayane et al. [34]. It is a multicomponent reaction of equimolar amounts of arylaldehyde, malononitrile and *β*-naphthol at the reflux of a hydroalcoholic solution in the presence of CuI as a catalyst. Their structures were confirmed on the basis oftheir ^1^H and ^13^C NMR spectra and by comparison with literature data [35].

**Scheme 2:**
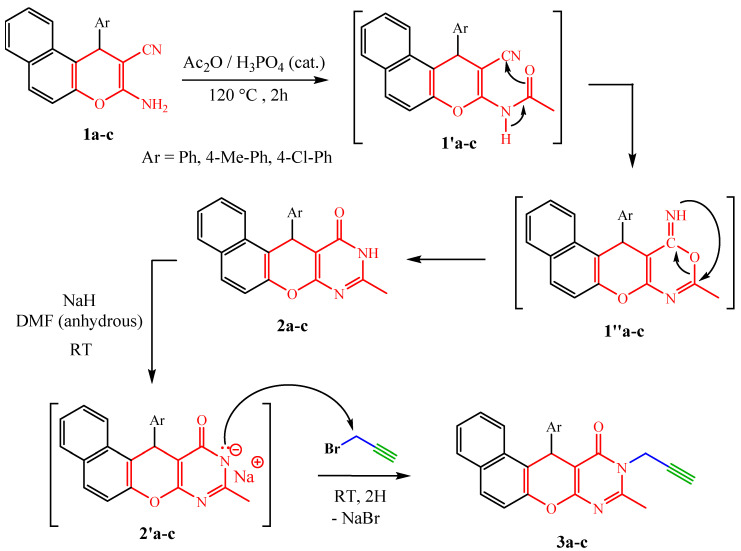
Synthetic pathway to compounds **2a**–**c** and **3a**–**c**.

Condensation reaction, of the last precursors, with acetic anhydride in the presence of polyphosphoric acid (PPA) under reflux for 2 h gives pyrimidinones **2a**–**c** in yields ranging from 66% to 80%. From a mechanistic point of view, the reaction is carried out in two steps. The precursors **1a**–**c** react with acetic anhydride to give the non-isolable intermediates **1′a**–**c** which cyclize to **1″a–c** after nucleophilic attack at the nitrile group. Dimroth-type intramolecular rearrangement [32,36] of the intermediate thus formed leads to pyranopyrimidinones **2a**–**c**, which reacted with propargyl bromide in the presence of sodium hydride in anhydrous DMF at room temperature to give the corresponding *N*-propargylated dipolarophiles **3a**–**c** in good yields (80–92%) (Scheme 2) [37].

The formed *N*-propargylatednaphthopyranopyrimidinones **3a**–**c** were identified on the basis of their ^1^H NMR and ^13^C NMR spectra. The ^1^H NMR spectra of these dipolarophiles allows to see, in addition to the signals of the protons introduced by the naphthopyranopyrimidinones **2a**–**c**, the disappearance of a singlet of the NH proton at δ_H_ 12.60–12.61 and the appearance of three new signals, two doublets of doublets are observed at δ_H_ 4.58–4.61 and 5.08–5.12 (*J* = 15.9, 2.4 Hz) which were attributed to methylenic protons (CH_2_) and a characteristic triplet of an acetylenic proton observed at δ_H_ 2.29–2.33 (*J* = 2.4 Hz). The proximity of methylene to the nitrogen atom bearing four different substituents (hybridized sp3) including its free doulet perfectly conjugated with the carbonyl of pyrimidinone is at the origin of the non-isochrony, and thus the non-equivalence of the methylenic protons which logically appear at two different chemical shifts. Moreover, the ^13^C NMR spectra of these derivatives exhibited the appearance of new signals at δ_C_ 31.7–32.7, 71.8–72.4 and 75.3–76.1 relative to new carbons C_4′_, C_5′_ and C_6′_introduced by the propargyl moiety, respectively.

**Scheme 3:**
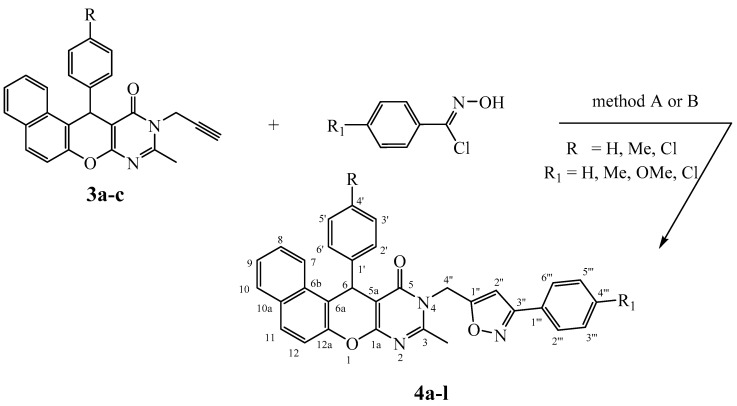
Synthesis of 3,5-isoxazole derivatives **4a–l**.

In order to access, via 1,3-dipolar cycloaddition reaction as shown in Scheme 3, to the new expected hybrid molecules **4a–l**, the *N*-propargylated derivatives **3a**–**c** were treated with various arylnitrile oxides, under conventional heating in refluxing DMF for 8–12 h (Method A) or under microwave irradiation in DMF for 3 to 5 min (Method B), both in the presence of copper(I) catalyst and Et_3_N as a base.

Until now, copper catalyzed nitrile oxide-alkyne click chemistry (CuAAC) is one of the methods of choice for regiospecific access to 3,5-disubstituted isoxazole derivatives [38].

**Scheme 4:**
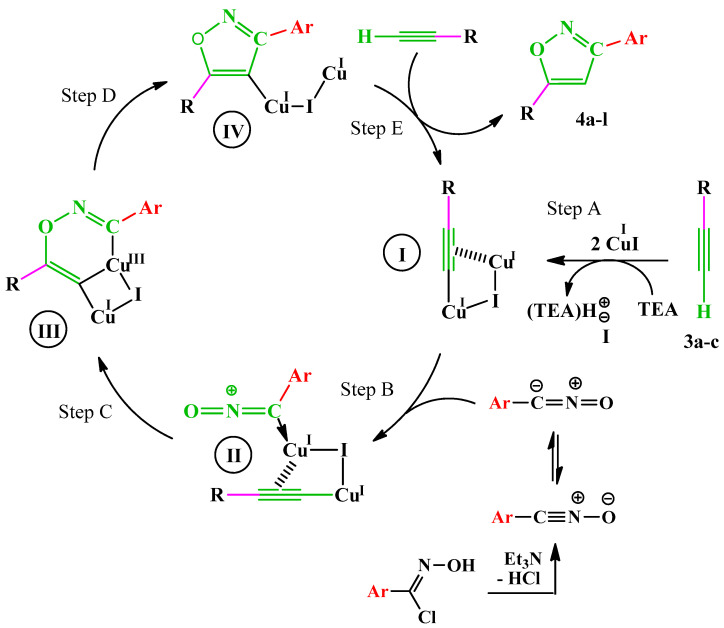
Proposed mechanism for the formation of compounds **4a–l**.

From a mechanistic point of view, the formation of copper(I) acetylide I (step A) occurs through a π-alkyne copper complex intermediate. The π-coordination of alkyne to copper significantly acidifies terminal hydrogen of the alkyne, bringing it into the proper range to be deprotonated in presence of TEA and resulting in the formation of a σ-acetylide with a second copper(I) atom. The added aryl nitrile oxide is then activated by coordination to copper (step B), thus forming the intermediate II. In the next step (C), the formation of the first C–O bond takes place, and a strained copper metallacycle III forms. This intermediate undergoes a rearrangement to lead to copper(I) isoxazole intermediate IV (step D), which in the presence of another molecule of alkyne gives the desired isoxazole while regenerating intermediate I (step E) (Scheme 4).

This reaction sequence generates the new hybrid molecules **4a–l** in moderate (method A/58–69%) to excellent yields (method B/91–97%). The absence of any other compound in the reaction mixture implies the strong regiospecificity of this reaction.

These resulting 3,5-regioisomers were confirmed from the NOE H-5_isoxazole_/H_methylene_ and H-5_isoxazole_/H_arom_, and the non-observation of any NOE H_methylene_/H_arom_. Thus, this 3,5-regiospecificity was explained by the uses of copper(I) catalyst.

The comparison of the obtained results shown in Table 1 from the conventional heating method (method A) and those from the MW-assisted synthesis way (method B) showed in all cases much shorter reaction times and higher yields were achieved under MW irradiation compared to classical heating.

The structures of the newly prepared compounds **4a–l** were raised on the basis of their spectroscopic measurements. In fact, compared to compounds **3**, the ^1^H NMR spectra of isoxazoles derivatives **4a–l** indicate the disappearance of the signal at δ_H_ 2.30–2.32 relative to the terminal acetylenic proton and the appearance of a new singlet at δ_H_ 6.50–6.51 (s, 1H) attributable to H_2′_ of the isoxazole ring, in addition to signals at δ_H_ 6.92–7.98 attributable to the protons of the aromatic moiety linked to the isoxazole system. Moreover, the ^13^C NMR spectra reinforced the structures of **4a–l** by showing the signals of the formed isoxazole moiety, as well as those from the dipolarophiles **3a**–**c**.

The ES-HRMS of compounds **3** and **4** showed the correct protonated molecular ion peaks [M+H]^+^ that complied with the proposed structures.

### 2.2. Molecular Docking Study

By studying the binding pocket of the SARS-CoV-2 M^pro^, it was found that the co-crystallized native inhibitor (N3) is located inside the pocket of the main protease receptor asymmetrically. The N3 ligand is a designed inhibitor for SARS-CoV-2 M^pro^ that was built from amino acids based on the M^pro^ pocket amino acids and cannot used medicinally. The main residues in this pocket were HIS-41, MET-49, PHE-140, GLY-143, HIS-164, MET-165, GLU-166, LEU-167, PRO-168, HIS-172, GLN-189, THR-190 and ALA-191.

Molecular docking of the synthesized molecules (**4a–l**) and N3 inhibitor into the main protease binding site was performed. The binding energies and interaction details (number of interactions, number of interacting amino acids, interacting amino acids and hydrogen bonds) of ligands with the target enzyme are presented in Table 2.

All compounds were perfectly placed in the active site by variable scores and binding interactions with the amino acids of the receptor pocket. From the docking results (Table 2), All the tested compounds (**4a–l**) achieved promising binding scores ranging from −7.6 to −8.9 kcal/mol, compared to the docked co-crystallized N3 inhibitor with a binding score of −7.0 kcal/mol. The synthesized compounds (**4a–l**) showed a much higher binding affinity towards M^pro^ enzyme (PDB: 6LU7) than N3.

It is worth mentioning that, especially for compounds **4a**, **4e** and **4i**, the obtained binding modes were quite similar to that of the docked N3 inhibitor, as depicted in Table 3. Moreover, these three selected compounds exhibited binding scores of −8.4, −8.5 and −8.5 kcal/mol, respectively, higher than of the docked co-crystallized N3 inhibitor (−7.0 kcal/mol) (Table 2).

The docked N3 inhibitor was stabilized inside the SARS-CoV-2 M^pro^ pocket through a three hydrogen bonds formation with GLY-143, HIS-164 and GLN-198 amino acids with the bond lengths of 2.96, 2.67 and 3.25 Å, respectively. Additionally, it formed a Pi-alkyl bond with MET-165 (bond length: 5.05 Å) and two alkyl interactions with HIS-41 (bond length: 4.28 Å) and MET-49 (bond length: 4.45 Å) residues. On the other side, the compound **4a** fitted inside the binding site of the SARS-CoV-2 M^pro^ by two hydrogen bonds formation, with GLY-143 and GLU-166 at 3.37 and 3.05 Å, respectively, with an additional carbon hydrogen bond with ASN-142 residue (bond length: 3.38 Å) and two Pi-sulfur interactions with MET-49 (bond length: 5.38 Å) andMET-165(bond length: 5.23 Å). Moreover, the compound **4e** showed a hydrogen bond with the GLY-143 amino acid at 3.19 Å. Additionally, it showed a carbon hydrogen bond with ASN-142 residue (bond length: 3.39 Å) and two Pi-sulfur interactions with MET-49 (bond length: 5.11 Å) and MET-165 (bond length: 5.75 Å). The compound **4i** formed a hydrogen bond, a carbon hydrogen bond and two Pi-sulfur interactions with the previously mentioned four amino acids as well. It formed a hydrogen bond with GLY-143 at 3.21 Å, a carbon hydrogen bond with ASN-142 at 3.46 Å and two Pi-sulfur interactions with MET-49 and MET-165 at 5.06 and 5.81 Å, respectively.

Although compounds **4k**, **4h** and **4j** did not exhibit very comparable interactions to those of N3 towards SARS-CoV-2 M^pro^, they, on the other hand, exhibited lower binding energy (−8.9, −8.5 and −8.4 kcal/mol, respectively) (Table 2). This finding can be explained, in part, by the nature of interactions between these compounds and the amino acids that constitute SARS-CoV-2 M^pro^ (Table 4). Indeed, the compound **4k** formed a hydrogen bond with THR-26 (bond length: 3.19 Å), three Pi-alkyl bonds with MET-49 (bond length: 5.39 Å), MET-165 (bond length: 4.82 Å) and PRO-168 (bond length: 4.53 Å), a Pi-sulfur interaction with CYS-145 (bond length: 5.10 Å) and a Pi-sigma interaction with HIS-41 (bond length: 3.71 Å). Moreover, the derivative **4h**, was engaged in a hydrogen bond, two Pi-donor hydrogen bonds, a Pi-sulfur interaction, two alkyl interactions and a Pi-alkyl bond with residues GLY-143 (bond length: 2.96 Å), ASN-142 (bond length: 3.65 Å), GLY-143 (bond length: 3.57 Å), CYS-145 (bond length: 4.79 Å), HIS-41 (bond length: 4.09 Å), CYS-145 (bond length: 4.48 Å) and PRO-168 (bond length: 4.88 Å), respectively. The compound **4j** interacts with in many amino acids of the main protease of COVID-19 (M^pro^) via two Hydrogen Bond, Pi-sigma interaction and a two Pi-alkyl bond with the side chain residues GLY-143 (bond length: 3.19 Å), GLU-166 (bond length: 3.15 Å), MET- 49 (bond length: 3.95 Å), CYS-145 (bond length: 5.25 Å), HIS-163 (bond length: 4.98 Å), respectively.

It is important to mention that the nature of the substituents allows the molecule to be placed into space in a well-defined conformation. Indeed, these substituents with different sizes promote or disadvantage possible free rotations, they also increase or decrease the electron density of certain groups essentially the aromatic ones by their indicative and/or mesomeric electronic effects. The spatial arrangement of the two benzene rings in compounds **4a–1** depends on the nature of their substituents (R and R_1_). This arrangement can, in certain cases, inhibit the delocalization of the electronic doublets by mainly mesomeric effects towards the outside of the ring (case of the aromatic system carried by the isoxazole nucleus), which influences the interaction of these aromatic systems with certain amino acids. Moreover, the R and R_1_ substituents do not need to engage in direct interactions with amino acids, but their sizes and electronic effects require special interactions of the corresponding aromatic rings with special amino acids. As mentioned in Table 3, the benzene ring at position C-6 of the pyran moiety and that carried by isoxazole both unsubstituted in **4a** exhibit Pi-sulfur interactions with MET-49 (bond length: 5.38 Å) andMET-165 (bond length: 5.23 Å), respectively. The same observation was noted with compounds **4e** and **4i** where the aromatic ring carried by the pyran is *para*-methylated and *para*-chlorinated, respectively and the benzene ring carried by the isoxazole in both dervivatives is unsubstituted. This result can be explained by the effect of the size of the methyl group and the chlorine atom on the spatial arrangement of the aromatic ring throughout the molecule. In addition, the inductive donor effect (+ I) exerted by the methyl group and the two opposite electronic effects (-I) and (+ M) exerted by the chlorine atom appear to lead to practically the same result. It was observed that in the case of compound **4h**, the benzenic nucleus attached to an isoxazole carrying in *para* position a chlorine atom (R_1_) exerts two Pi-donor Hydrogen bonds interactions with ASN-142 (bond length: 3.65 Å). These types of interactions were not observed in the rest of the products with other R and R_1_ which proves that the nature of these two substituents give the entire molecule a well-defined conformation which also guarantees well-defined interactions. With R = Cl and R_1_ = OMe, the compound **4k**, takes a new conformation which allows, among other things, the OMe group to engage in a hydrogen bond with THR-26 (bond length: 3.19 Å).

According to our findings, the tested compounds, in particular **4a**, **4e**, **4h**, **4i**, **4j** and **4k**, could effectively act as novel antiviral agents against SARS-CoV-2.

## 3. Methods and Materials

### 3.1. Chemistry

#### 3.1.1. General Experimental Procedures

All synthesis reactions were checked by TLC using aluminium sheets of silica gel 60 F_254_, 0.2 mm. Melting points were measured on an electrothermal 9002 apparatus and were reported uncorrected. NMR spectra were run on a Bruker AC-300 spectrometer at 300 MHz (^1^H) and 75 MHz (^13^C). All chemical shifts were reported as δ values (ppm) relative to residual non-deuterated solvent. Mass spectra were recorded with HRMS-ES using the reflectron mode in the positive ion mode. Microwave-assisted synthesis was peformed in a Start Synth multimode microwave instrument producing controlled irradiation at 2.45 GHz (Milestone S.r.l., Sorisole, Italy). The device is equipped with an industrial magnetron and a microwave diffuser providing a continuous microwave output power from 0 to 1400 W. An open reaction vessel was employed in all reactions.

The starting materials **1** and **2** were prepared according to the literature [32].

#### 3.1.2. General Procedure for the Preparation of Naphthopyranopyrimidinone N-Propargylated Derivatives **3a**–**c**

1 mmol of naphthopyranopyrimdinone **2a**–**c** was dissolved in 15 mL of anhydrous DMF then 2 equivalents of sodium hydride (NaH) were added and the reaction mixture was stirred at room temperature under an argon atmosphere for 30 min; afterwards, 1.5 eq of propargyl bromide was added slowly, and the mixture was stirred for another 1.5 h under positive atmosphere of argon. Upon completion of the reaction, monitored by TLC, we added distilled water and the formed precipitate was collected by filtration, washed with water and purified by flash column chromatography on silica gel (EtOAc/PE, 1:1, as an eluent).

Compound **3a:** 3-methyl-6-phenyl-4-(prop-2-yn-1-yl)-10*H*-naphtho[2,1-*b*]pyrano[2,3-*d*]pyrimidin-11(12*H*)-one

Brown solid, yield 88%, mp 193–195 °C.^1^H NMR (300 MHz; CDCl*_3_*): δ(ppm) = 2.22 (s, 3H, H_9__′_); 2.32 (t, 1H, H_6′__’_, *J* = 2.4 Hz); 4.69 (dd, 1H, H_4′′a_, *J* = 2.4 Hz, *J* = 17.4 Hz); 5.12 (dd, 1H, H_4′′b_, *J* = 2.4 Hz, *J* = 17.4 Hz); 5.79 (s, 1H, H_6_); 7.03 (d, 2H, H_2′,6′_, *J* = 8.1 Hz); 7.43 (m, 6H, H_arom._); 7.79 (d, 2H, H_11,12_, *J* = 7.6 Hz); 7.92 (d, 1H, H_7_, *J* = 8.1 Hz). ^13^C NMR (75 MHz; CDCl*_3_*):δ(ppm) = 23.2, 32.7, 35.3, 71.9, 75.3, 102.2, 116.1, 117.0, 123.6, 124.4, 126.6, 127.7, 127.9, 128.6, 128.9, 130.5, 131.2, 135.8, 140.6, 147.5, 157.3, 158.9, 161.0. ESI-HRMS [M+H]^+^calcd. For (C_25_H_18_N_2_O_2_)^+^: 379.1446, found: 379.1456.

Compound **3b:** 3-methyl-4-(prop-2-yn-1-yl)-6-(*p*-tolyl)-10*H*-naphtho[2,1-*b*]pyrano[2,3-*d*]pyrimidin-11(12*H*)-one

Brown solid, yield 92%, mp 192–194 °C.^1^H NMR (300 MHz; CDCl*_3_*): δ(ppm) = 2.22 (s, 3H, H_9′_); 2.29 (t, 1H, H_6′__’_, *J* = 2.4 Hz); 2.67 (s, 3H, H_4′_); 4.58 (dd, 1H, H_4′′a_, *J* = 2.4 Hz, *J* = 17.4 Hz); 5.08 (dd, 1H, H_4′′b_, *J* = 2.4 Hz, *J* = 17.4 Hz); 5.87 (s, 1H, H_6_); 7.03 (d, 2H, H_3′,5′_, *J* = 8.1 Hz); 7.38 (m, 5H, H_aromatic_); 7.82 (d, 2H, H_11,12_, *J* = 7.4 Hz); 7.92 (d, 1H, H_7_, *J* = 8.1 Hz). ^13^C NMR (75 MHz; CDCl*_3_*):δ(ppm) = 20.4, 22.0, 32.7, 35.9, 72.4, 76.1, 101.1, 116.1, 116.9, 123.2, 124.4, 126.6, 127.8, 127.9, 128.6, 128.8, 130.5, 131.0, 135.8, 140.2, 147.5, 157.3, 158.9, 160.9. ESI-HRMS [M+H]^+^calcd. For (C_26_H_19_N_2_O_2_)^+^: 392.1524, found: 392.1531.

Compound **3c:** 6-(4′′-chlorophenyl)-3-methyl-4-(prop-2-yn-1-yl)-10*H*-naphtho[2,1-*b*]pyrano[2,3-*d*]pyrimidin-11(12*H*)-one

Brown solid, yield 80%, mp 194–196 °C.^1^H NMR (300 MHz; CDCl*_3_*): δ(ppm) = 2.24 (s, 3H, CH_3_); 2.33 (t, 1H, H_6′__’_, *J* = 2.4 Hz); 4.62 (dd, 1H, H_4′′_, *J* = 2.4 Hz, *J* = 17.4 Hz); 5.18 (dd, 1H, H_4′′_, *J* = 2.4 Hz, *J* = 17.4 Hz); 5.85 (s, 1H, H_6_); 7.05 (d, 2H, H_2′,6′_, *J* = 8.1 Hz); 7.40 (m, 5H, H_aromatic_); 7.82 (d, 2H, H_11,12_, *J* = 7.4 Hz); 7.90 (d, 1H, H_7_, *J* = 8.1 Hz). ^13^C NMR (75 MHz; CDCl*_3_*):δ(ppm) = 22.6, 31.7, 35.4, 71.8, 75.4, 102.4, 116.1, 116.9, 123.2, 124.4, 126.6, 127.8, 127.9, 128.7, 128.9, 130.4, 131.0, 136.8, 140.4, 146.5, 156.3, 157.9, 161.3. ESI-HRMS [M+H]^+^calcd. For (C_25_H_16_ClN_2_O_2_)^+^: 412.0978, found: 412.0989.

#### 3.1.3. General Procedure for the Preparation of Isoxazoles Derivatives **4a–l**

Method A (conventional method): Alkynes **3a**–**c** (1 mmol) and appropriate hydroximyl chloride (2 mmol) were dissolved in 3 mL DMF. Cuprous iodide (I) (0.1 equiv) was added with stirring followed by Et_3_N (0.4 mmol) at room temperature. The mixture was refluxed for 8–12 h. After cooling, the mixture was diluted with water and then extracted with ethyl acetate (3 × 30 mL). The organic layer was dried over Na_2_SO_4_. After the removal of solvent in vacuo, the resulting residue was purified by silica gel column chromatography eluted with dichloromethane to obtain the desired new products (**4a–l**).

Method B (microwave-assisted): Alkynes **3a**–**c** (1 mmol) and appropriate hydroximyl chloride (2 mmol) were dissolved in 3 mL DMF. Cuprous iodide (I) (0.1 equiv) was added with stirring followed by Et_3_N(0.4 mmol) at room temperature. The reaction mixture was then subjected to microwave irradiations at 250 Watts for 3–5 min, after which it was diluted with water and then extracted with ethyl acetate (3 × 30 mL). The organic layer was dried over Na_2_SO_4_. After removal of solvent under reduced pressure, the resulting residue was purified by silica gel column chromatography eluted with dichloromethane to abtain the desired new products (**4a–l**).

Compound **4a:** 3-methyl-6-phenyl-4-((3′′-phenylisoxazol-5”-yl)methyl)-10*H*-naphtho[2,1-*b*]pyrano[2,3-*d*]pyrimidin-11(12*H*)-one

White solid, yield 97%, mp 148–150 °C.^1^H NMR (300 MHz; CDCl*_3_*): δ (ppm) = 2.72 (s, 3H, H_9′_); 5.13 (d, 1H, H_4′′_, *J* = 15.9 Hz); 5.57 (d, 1H, H_4′′_, *J* = 15.9 Hz); 5.87 (s, 1H, H_6_); 6.54 (s, 1H, H_2′′_);7.19 (d, 2H, H_2′′’_,_6′′’_, *J* = 8.4 Hz); 7.26–7.49 (m, 9H, H_arom_); 7.70–7.72 (m, 2H, H_arom_);7.79–7.83 (m, 3H, H_arom_). ^13^C NMR (75 MHz; CDCl*_3_*): δ (ppm) = 22.4, 35.7, 39.2, 100.7, 101.7, 115.2, 116.9, 122.9, 124.6, 126.3, 126.7, 127.8, 128.0, 128.4, 129.2, 129.3, 129.7, 130.3, 131.1, 132.1, 141.5, 147.5, 157.6, 161.3, 162.4, 165.6. ESI-HRMS [M+H]^+^ calcd. For (C_32_H_23_N_3_O_3_)^+^: 498.1817, found: 498.1822.

Compound **4b:** 3-methyl-6-phenyl-4-((3′′-(*p*-tolyl)isoxazol-5”-yl)methyl)-10*H*-naphtho[2,1-*b*]pyrano[2,3-*d*]pyrimidin-11(12*H*)-one

White solid, yield 93%, mp 138–140 °C. ^1^H NMR (300 MHz; CDCl*_3_*): δ (ppm) = 2.36 (s, 3H, H_9′_); 2.73 (s, 3H, H_7′′’_); 5.11 (d, 1H, H_4′′_, *J* = 15.9 Hz); 5.61(d, 1H, H_4′′_, *J* = 15.6 Hz); 5.92 (s, 1H, H_6_); 6.54 (s, 1H, H_2′′_);7.15 (d, 2H, H_2′_*,*_6′_, *J* = 7.2 Hz); 7.21–7.28 (m,5H, H_arom_); 7.35–7.53 (m, 3H, H_arom_); 7.61 (d, 2H, H_arom_, *J* = 8.1 Hz); 7.83 (m, 2H, H_arom_); 7.97 (d, 1H, H_11_, *J* = 7.8 Hz). ^13^C NMR (75 MHz; CDCl*_3_*): δ (ppm) = 21.3, 22.9, 36.8, 39.7, 101.7, 102.1, 116.3, 117.4, 123.6, 125.0, 125.5, 126.7, 126.8, 127.1, 128.4, 128.5, 128.5, 129.5, 131.0, 140.4, 143.6, 148.0, 157.9, 159.5, 161.8, 162.8, 166.0. ESI-HRMS [M+H]^+^ calcd. For (C_33_H_25_N_3_O_3_)^+^: 512.1973, found: 512.1988.

Compound **4c:** 4-((3′′-(*p*-methoxyphenyl)isoxazol-5”-yl)methyl)-3-methyl-6-phenyl-10*H*-naphtho[2,1-*b*]pyrano[2,3-*d*]pyrimidin-11(12*H*)-one

White solid, yield 92%, mp 166–168 °C. ^1^H NMR (300 MHz; CDCl*_3_*): δ (ppm) = 2.71 (s, 3H, H_9′_); 3.84 (s, 3H, H_7′′’_); 5.07 (d, 1H, H_4′′_, *J* = 15.6 Hz); 5.58 (d, 1H, H_4′′_, *J* = 15.9Hz); 5.91 (s,1H, H_6_);6.50 (s,1H, H_2′′_); 6.92 (d, 2H, H_3′′’,5′′’_, *J* = 9.6 Hz); 6.96 (d, 1H, H_arom_, *J* = 7.6 Hz); 7.12 (m, 2H, H_arom_); 7.41–7.48 (m, 5H, H_arom_); 7.64 (d, 2H, H_2′′’,6′′’_, *J* = 9.6 Hz); 7.68 (m,2H, H_arom_); 7.69 (d, 1H, H_1_, *J* = 7.4 Hz). ^13^C NMR (75 MHz; CDCl*_3_*): δ (ppm) = 22.4, 36.3, 39.2, 54.8, 101.1, 101.5, 113.8, 115.8, 116.9, 120.3, 123.1, 124.5, 126.2, 126.6, 127.7, 127.9, 128.0, 129.0, 130.5, 131.1, 143.1, 147.5, 157.4, 158.9, 160.6, 161.3, 161.9, 165.4. ESI-HRMS [M+H]^+^ calcd. For (C_33_H_25_N_3_O_4_)^+^: 528.1923, found: 528.1935.

Compound **4d:** 4-((3′′-(*p*-chlorophenyl)isoxazol-5”-yl)methyl)-3-methyl-6-phenyl-10*H*-naphtho[2,1-*b*]pyrano[2,3-*d*]pyrimidin-11(12*H*)-one

White solid, yield 92%, mp 150–152 °C. ^1^H NMR (300 MHz; CDCl*_3_*): δ (ppm) = 2.73 (s, 3H, H_9′_); 5.12 (d, 1H, H_4′′_, *J* = 15.6 Hz); 5.60 (d, 1H, H_4′′_, *J* = 15.9 Hz); 5.91 (s,1H, H_6_); 6.54 (s,1H, H_2′′_); 7.10 (m, 1H, H_arom_); 7.28 (m, 2H, H_arom_); 7.37–7.49 (m, 7H, H_arom_); 7.66 (d, 2H, H_2′′’,6′′’_, *J* = 8.7 Hz); 7.67 (m,2H, H_arom_); 7.96 (d, 1H, H_11_, *J* = 7.8 Hz).^13^C NMR (75 MHz; CDCl*_3_*): δ (ppm) = 22.9, 36.8, 39.7, 101.7, 102.1, 116.3, 117.4, 123.6, 125.0, 126.7, 126.8, 127.1, 128.0, 128.4, 128.5, 128.6, 129.2, 129.5, 131.0, 131.6, 136.3, 143.6, 148.0, 157.8, 159.5, 161.8, 161.9, 166.6. ESI-HRMS [M+H]^+^ calcd. For (C_32_H_22_ClN_3_O_3_)^+^: 532.1427, found: 532.1437.

Compound **4e:** 3-methyl-4-((3′′-phenylisoxazol-5”-yl)methyl)-6-(*p*-tolyl)-10*H*-naphtho[2,1-*b*]pyrano[2,3-*d*]pyrimidin-11(12*H*)-one

White solid, yield 95%, mp 157–159 °C. ^1^H NMR (300 MHz; CDCl*_3_*): δ (ppm) = 2.19 (s, 3H, H_9′_); 2.73 (s, 3H, H_7′_); 5.08 (d, 1H, H_4′′_, *J* = 15.6 Hz); 5.63 (d, 1H, H_4′′_, *J* = 15.6 Hz); 5.88 (s,1H, H_6_);6.55 (s,1H, H_2′′_); 7.05 (d, 2H, H_2′′’,6′′’_, *J* = 7.8 Hz); 7.14–7.34 (m, 3H, H_arom_);7.37–7.57 (m, 5H, H_arom_); 7.66 (d, 2H, H_3′′’,5′′’_, *J* = 7.8 Hz); 7.83 (d, 2H, H_5_,_6_, *J* = 9.0 Hz); 7.98 (d, 1H, H_1_, *J* = 8.1 Hz).^13^C NMR (75 MHz; CDCl*_3_*): δ (ppm) = 21.4, 22.9, 36.3, 39.7, 101.2, 101.6, 115.9, 116.8, 123.1, 124.5,126.3, 126.5, 127.5, 127.8, 128.0, 128.6, 128.7, 128.9, 130.5, 135.8, 135.9, 140.2, 147.4, 157.2, 158.9, 161.3, 161.4, 166.1. ESI-HRMS [M+H]^+^ calcd. For (C_33_H_25_N_3_O_3_)^+^: 512.1974, found: 512.1981.

Compound **4f:** 3-methyl-6-(*p*-tolyl)-4-((3′′-(*p*-tolyl)isoxazol-5”-yl)methyl)-10*H*-naphtho[2,1-*b*]pyrano[2,3-*d*]pyrimidin-11(12*H*)-one

White solid, yield 96%, mp 154–156 °C. ^1^H NMR (300 MHz; CDCl*_3_*): δ (ppm) = 1.72 (s, 3H, H_9′_); 2.23 (s, 3H, H_7′_); 2.72 (s, 3H, H_7′′’_); 5.10 (d, 1H, H_4′′_, *J* = 15.9 Hz); 5.63 (d, 1H, H_4′′_, *J* = 15,9 Hz); 5.89 (s, 1H, H_6_); 6.58 (s, 1H, H_2′′_);7.05 (d, 2H, H_3′′’_,_5′′’_, *J* = 7.8 Hz); 7.26–7.49 (m, 6H, H_arom_); 7.61–7.83 (m, 5H, H_arom_); 7.98 (d, 1H, H_1_, *J* = 8.1 Hz). ^13^C NMR (75 MHz; CDCl*_3_*): δ (ppm) = 20.9,22.9, 36.3, 39.2, 101.8, 102.2, 116.5, 117.4, 123.6, 125.0, 126.7, 126.8, 127.1, 128.3, 128.4, 128.5, 128.8, 128.9, 129.1, 129.4, 130.2, 131.0, 131.6, 136.3, 140.7, 148.0, 157.7, 159.4, 161.8, 162.8, 166.3. ESI-HRMS [M+H]^+^ calcd. For (C_34_H_27_N_3_O_3_)^+^: 526.2130, found: 536.2137.

Compound **4g:** 4-((3′′-(*p*-methoxyphenyl)isoxazol-5”-yl)methyl)-3-methyl-6-(*p*-tolyl)-10*H*-naphtho[2,1-*b*]pyrano[2,3-*d*]pyrimidin-11(12*H*)-one

White solid, yield 94%, mp 144–146 °C. ^1^H NMR (300 MHz; CDCl*_3_*): δ (ppm) = 2.23 (s, 3H, H_9′_); 2.72 (s, 3H, H_7′_); 3.85 (s, 3H, H_7′′’_); 5.06 (d, 1H, H_4′′_, *J* = 15.6 Hz); 5.61 (d, 1H, H_4′′_, *J* = 15.9 Hz); 5.88 (s,1H, H_6_);6.52 (s, 1H, H_2′′_); 6.92 (d, 2H, H_3′′’,5′′’_, *J* = 8.7 Hz); 6.96 (d, 2H, H_arom_, *J* = 7.4 Hz); 7.02–7.48 (m, 5H, H_arom_); 7.65 (d, 2H, H_2′′’,6′′’_, *J* = 8.7 Hz); 7.82 (m, 2H, H_arom_); 7.98 (d, 1H, H_11_, *J* = 8.4 Hz). ^13^C NMR (75 MHz; CDCl*_3_*): δ (ppm) = 20.9, 22.9, 36.3, 39.7, 55.3, 101.8, 102.0, 114.3, 116.5, 117.4, 120.8, 123.6, 124.9, 127.1,128.2, 128.3, 128.4, 129.1, 129.3, 131.0, 131.5, 136.3, 140.7, 148.0, 157.8, 159.4, 161.1, 161.8, 162.4, 166.0. ESI-HRMS [M+H]^+^ calcd. For (C_34_H_27_N_3_O_4_)^+^: 542.2079, found: 542.2088.

Compound **4h:** 4-((3-(4-chlorophenyl)isoxazol-5-yl)methyl)-3-methyl-6-(*p*-tolyl)-10*H*-naphtho[2,1-*b*]pyrano[2,3-*d*]pyrimidin-11(12*H*)-one

White solid, yield 97%, mp 140–142 °C. ^1^H NMR (300 MHz; CDCl*_3_*): δ (ppm) = 2.23 (s, 3H, H_9′_); 2.72 (s, 3H, H_7′_); 5.09 (d, 1H, H_4′′_, *J* = 15.9 Hz); 5.62 (d, 1H, H_4′′_, *J* = 15.9 Hz); 5.88 (s,1H, H_6_);6.56 (s,1H, H_2′′_); 7.03 (d, 2H, H_2′′’,6′′’_, *J* = 8.4 Hz); 7.37 (m, 2H, H_arom_);7.39–7.48 (m, 5H, H_arom_); 7.65 (d, 2H, H_3′′’,5′′’_,*J* = 8.4 Hz); 7.67 (m,2H, H_arom_); 7.69 (d, 1H, H_11_, *J* = 7.8 Hz). ^13^C NMR (75 MHz; CDCl*_3_*): δ (ppm) = 21.3, 22.9, 36.3, 39.7, 101.8, 102.1, 116.5, 117.4, 123.6, 124.9, 125.5, 126.6, 126.7, 127.1, 128.3, 128.4, 129.1, 129.3, 129.4, 129.6, 131.0, 131.5, 136.3, 140.4, 140.7, 148.0, 157.8, 166.1. ESI-HRMS [M+H]^+^ calcd. For (C_33_H_24_ClN_3_O_3_)^+^: 546.1584, found: 546.1594.

Compound **4i:** 6-(4-chlorophenyl)-3-methyl-4-((3′′-phenylisoxazol-5”-yl)methyl)-10*H*-naphtho[2,1-*b*]pyrano[2,3-*d*]pyrimidin-11(12*H*)-one

White solid, yield 93%, mp 122–124 °C. ^1^H NMR (300 MHz; CDCl*_3_*): δ (ppm) = 2.71 (s, 3H, H_9′_); 5.09 (d, 1H, H_4′′_, *J* = 15.9 Hz); 5.59 (d, 1H, H_4′′_, *J* = 15.9 Hz); 5.92 (s, 1H, H_6_); 6.57 (s, 1H, H_2′′_);7.14 (d, 2H, H_2′′’,6′′’_, *J* = 7.5 Hz); 7.14–7.23 (m, 2H, H_arom_); 7.26–7.49 (m, 9H, H_arom_); 7.59–7.72 (m, 2H, H_arom_); 7.49–7.57 (m, 2H, H_arom_); 7.74 (d, 1H, H_1_, *J* = 7.6 Hz). ^13^C NMR (75 MHz; CDCl*_3_*): δ (ppm) = 22.4,36.3, 39.2, 101.2, 101.7, 115.8, 116.9, 123.1, 124.5, 126.3, 127.8, 127.9, 128.0, 128.4, 129.0, 129.7, 130.5, 131.1, 143.1, 147.5, 157.4, 159.0, 161.3, 162.3, 165.8. ESI-HRMS [M+H]^+^ calcd. For (C_32_H_23_ClN_3_O_3_)^+^: 532.1427, found: 532.1435.

Compound **4j:** 6-(*p*-chlorophenyl)-3-methyl-4-((3′′-(*p*-tolyl)isoxazol-5”-yl)methyl)-10*H*-naphtho[2,1-*b*]pyrano[2,3-*d*]pyrimidin-11(12*H*)-one

White solid, yield 91%, mp 147–149 °C. ^1^H NMR (300 MHz; CDCl*_3_*): δ (ppm) = 2.39 (s, 3H, H_9′_); 2.74 (s, 3H, H_7′′’_); 5.15 (d, 1H, H_4′′_, *J* = 15.9Hz,); 5.59 (d, 1H, H_4′′_, *J* = 15.6Hz); 5.89 (s,1H, H_6_);6.54 (s,1H, H_2′′_); 7.18–7.28 (m, 4H, H_arom_); 7.36–7_._49 (m, 5H, H_arom_);7.64 (d, 2H, H_arom_, *J* = 8.1 Hz); 7.89 (m, 3H, H_arom_).^13^C NMR (75 MHz; CDCl*_3_*): δ (ppm) = 21.4, 23.0, 36.2, 39,7, 101.5, 102.2, 114.8, 116.5, 117.4, 123.6, 124.9, 125.5, 126.6, 126.7, 127.1, 128.3, 128.4, 129.1, 129.3, 129.4, 129.6, 131.0, 131.5, 136.3, 140.4, 140.7, 148.0, 157.8, 166.1. ESI-HRMS [M+H]^+^ calcd. For (C_33_H_24_ClN_3_O_3_)^+^: 546.1584, found: 546.1594.

Compound **4k:** 6-(*p*-chlorophenyl)-4-((3′′-(*p*-methoxyphenyl)isoxazol-5”-yl)methyl)-3-methyl-10*H*-naphtho[2,1-*b*]pyrano[2,3-*d*]pyrimidin-11(12*H*)-one

White solid, yield 96%, mp 160–162 °C. ^1^H NMR (300 MHz; CDCl*_3_*): δ (ppm) = 2.74 (s, 3H, H_9′_); 3.80 (s,3H, H_7′′’_); 5.14 (d, 1H, H_4′′_, *J* =15.9 Hz); 5.58 (d, 1H, H_4′′_, *J* = 16.5 Hz); 5.89 (s, 1H, H_6_); 6.50 (s, 1H, H_2′′_);6.97 (d, 2H, H_3′′’,5′′’_, *J* = 8.7 Hz); 7.18 (d, 2H, H_2′,6′_, *J* = 8.4 Hz); 7.36 (d, 2H, H_2′′’,6′′’_, *J* = 8.7 Hz); 7.26–7.49 (m,4H, H_arom_); 7.70 (d, 2H, H_arom_, *J* = 7.0 Hz); 7.73–7.89 (m, 3H, H_arom_). ^13^C NMR (75 MHz; CDCl*_3_*): δ (ppm) = 22.7,36.2, 38.7, 55.8, 101.1, 101.5, 113.8, 115.8, 116.9, 120.3, 123.1, 124.5, 126.2, 126.6, 127.7, 127.9, 128.0, 129.0, 130.5, 131.1, 143.1, 147.5, 157.4, 158.9, 160.6, 161.3, 161.9, 165.4. ESI-HRMS [M+H]^+^ calcd. For (C_33_H_25_ClN_3_O_4_)^+^: 562.1533, found: 562.1548.

Compound **4l:** 6-(*p*-chlorophenyl)-4-((3′′-(*p*-chlorophenyl)isoxazol-5”-yl)methyl)-3-methyl-10*H*-naphtho[2,1-*b*]pyrano[2,3-*d*]pyrimidin-11(12*H*)-one

White solid, yield 92%, mp 152–154 °C. ^1^H NMR (300 MHz; CDCl*_3_*): δ (ppm) = 2.74 (s, 3H, H_9′_); 5.16 (d, 1H, H_4′′_, *J* = 15.9 Hz); 5.58 (d, 1H, H_4′′_, *J* = 15.9 Hz); 5.89 (s, 1H, H_6_); 6.53 (s, 1H, H_2′′_); 7.18 (m, 2H, H_arom_); 7.20–7.69 (m, 8H, H_arom_); 7.81–7.83 (m, 2H, H_arom_); 7.85–7.89 (m, 2H, H_arom_). ^13^C NMR (75 MHz; CDCl*_3_*): δ (ppm) = 22.9, 36.8, 39.7, 101.2, 102.1, 115.6, 117.4, 123.4, 125.2, 126.8, 127.3, 127.7, 127.9, 128.0, 128.5, 128.6, 129.0, 129.1, 129.2, 129.3, 129.8, 130.8, 131.6, 132.6, 136.3, 142.0, 148.0, 158.0, 159.5, 161.7, 161.9, 166.5. ESI-HRMS [M+H]^+^ calcd. For (C_33_H_21_Cl_2_N_3_O_3_)^+^: 566.1038, found: 566.1050.

The NMR spectra of all synthesized compounds are given as supplementary material.

### 3.2. Molecular Docking Procedure

The chemical compound structures of the co-crystallized inhibitor (N3) and the synthesized compounds (**4a–l**) were generated and optimized using ACD (3D viewer) software (Version 2017.2.1, http://www.filefacts.com/acd3d-viewer-freeware-info, accessed on 8 July 2021), where their energies were minimized. The structure 6LU7 for RBVS (receptor-based virtual screening) having a catalytic dyad (Cys145 and His41), was selected because of its easy ability to complex with the co-crystallized peptide-like ligand inhibitor N3 used as a reference standard in the present work [28]. The crystal structure of SARS-CoV-2 M^pro^ (PDB: 6LU7) was obtained from the RSCB data bank (https://www.rcsb.org, accessed on 8 July 2021). The protein was prepared by removing the complexed inhibitor ligand and water molecules. Then, the polar hydrogens were added followed by appending Kollman charges. Hence, the grid box with dimensions of 40 × 40 × 40 points, spacing of 1.0 Å and centered with coordinates x: −11.993, y: 15.425, and z: 65.951, was generated based on N3 binding position in the target protein binding site. The molecular docking analysis of N3 and the synthesized compounds (**4a–l**) were performed using AutoDock Vina software (The Scripps Research Institute, La Jolla, CA, USA) [39]. Molecule–enzyme interactions were drawn and construed by employing the Biovia Discovery Studio Visualizer, BIOVIA, San Diego, CA, USA (2017).

## 4. Conclusions

In conclusion, we have successfully synthesized in this work a new series of 3,5-isoxazole linked naphthopyranopyrimidinones **4a–l**, in three steps, using the naphtopyranyl moiety as a building blocks support, via 1,3-dipolar cycloaddition reaction using arylnitrile oxides catalyzed by Cu(I) under classical and microwave irradiation conditions. The last condition offers several advantages including good-to-high yields, cleaner products, and a reduction in reaction time, making this method particularly attractive. In addition, to help fight COVID-19, molecular docking based on virtual screening was performed to identify among the newly synthesized compounds **4a–1** which ones has the potential to interact with COVID-19 M^pro^. Our results demonstrate that compounds **4a**, **4e**, **4h**, **4i**, **4j** and **4k**, exhibited good binding affinity towards the main protease of COVID-19 (M^pro^) better than that made by the native co-ligand N3 inhibitor. These molecules could possibly be promising in terms of therapeutic effect against SARs-CoV-2. However, in vitro and then in vivo investigation are essential to validate our prediction results and pave the way for the discovery of anti-SARs-CoV-2 drugs.

## Figures and Tables

**Table 1:** Comparison between the conventional heating procedure (method A) and microwave irradiation (method B) for the synthesis of compounds **4a–l**.

Product	R	R_1_	Time (h)	Yield (%) ^a^	Time (min)	Yield (%) ^b^
**4a**	H	H	8	69	5	97
**4b**	H	Me	12	63	3	93
**4c**	H	OMe	10	62	5	92
**4d**	H	Cl	8	60	5	92
**4e**	Me	H	12	67	3	95
**4f**	Me	Me	12	65	3	96
**4g**	Me	OMe	8	64	3	94
**4h**	Me	Cl	12	68	3	97
**4i**	Cl	H	9	62	5	93
**4j**	Cl	Me	8	58	3	91
**4k**	Cl	OMe	8	67	3	96
**4l**	Cl	Cl	12	60	3	92

^a^ Isolated yield after column chromatography. ^b^ Microwaveirradiation (250 W).

**Table 2:** Binding energy (kcal/mol) and interaction detail of compounds **4a–l** docked in the active site of M^pro^ enzyme (PDB: 6LU7).

Compound	Binding Energy (kcal/mol)	Interaction Detail: NI/NIAA: IAA
**4a**	−8.4	5/5: MET-49, ASN-142, GLY-143 *, MET-165, GLU-166 *
**4b**	−7.7	8/4: MET-165, GLU-166 *, PRO-168, ALA-191
**4c**	−7.7	7/5: MET-49, GLY-143 *, CYS-145, HIS-163, GLU-166
**4d**	−7.7	8/4: MET-165, GLU-166 *, PRO-168, ALA-191
**4e**	−8.5	4/4: MET- 49, ASN-142, GLY-143 *, MET-165
**4f**	−7.9	8/4: MET-165, GLU-166 *, PRO-168, ALA-191
**4g**	−7.6	6/5: MET- 49, GLY-143 *, CYS-145, HIS-163, GLU-166
**4h**	−8.5	7/5: HIS-41, ASN-142, GLY-143 *, CYS-145, PRO-168
**4i**	−8.5	4/4: MET- 49, ASN-142, GLY-143 *, MET-165
**4j**	−8.4	5/5: MET- 49, GLY-143 *, CYS-145, HIS-163, GLU-166 *
**4k**	−8.9	6/6: THR-26 *, HIS-41, MET-49, CYS-145, MET-165, PRO-168
**4l**	−7.7	7/3: MET-165, GLU-166 *, PRO-168
**N3**	−7.0	6/6: HIS-41, MET-49, GLY-143 *, HIS-164 *, MET-165, GLN-189 *

NI: number of interactions, NIAA: number of interacting amino acids, IAA: interacting amino acids, * = One hydrogen bond.

**Table 3:** 3D representations showing the binding interactions and positioning between the three promising tested compounds (**4a**, **4e** and **4i**) and the N3-binding pocket in comparison to the docked **N3** inhibitor.

Code	3D Binding Interactions	3D Pocket Positioning
**4a**	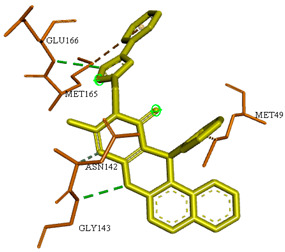	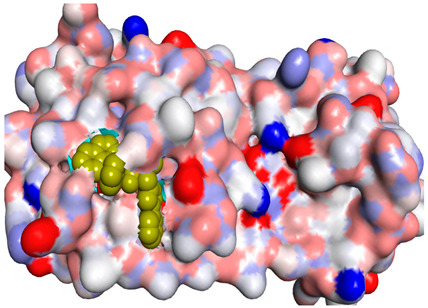
**4e**	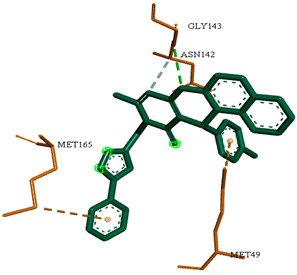	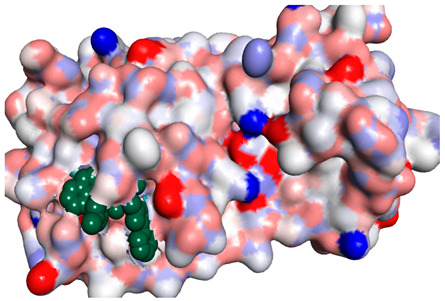
**4i**	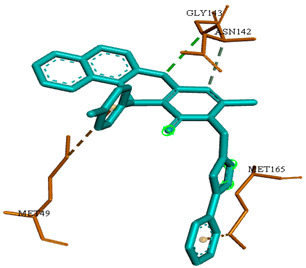	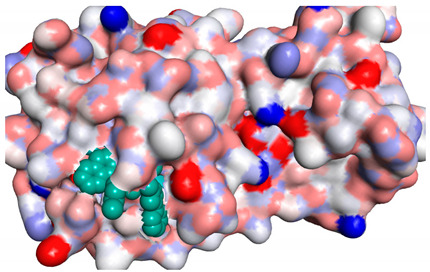
**N3**	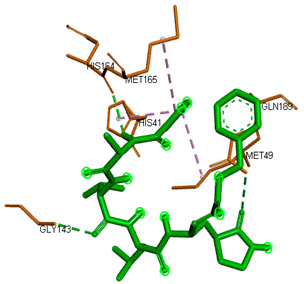	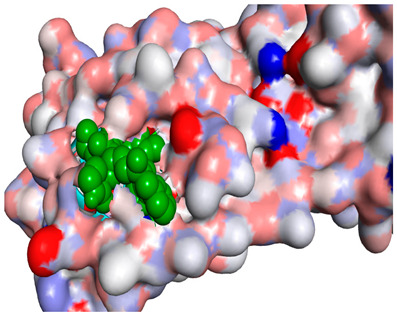

**Table 4:** 3D representations showing the binding interactions and positioning between the three promising tested compounds (**4k**, **4h** and **4j**) and the N3-binding pocket.

Code	3D Binding Interactions	3D Pocket Positioning
**4k**	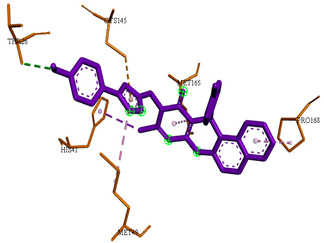	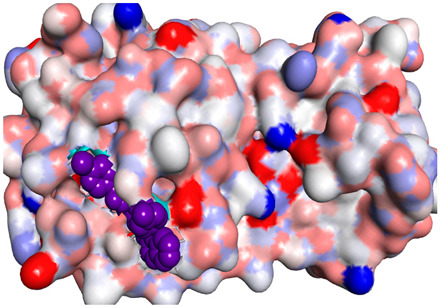
**4h**	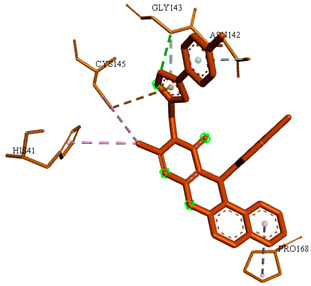	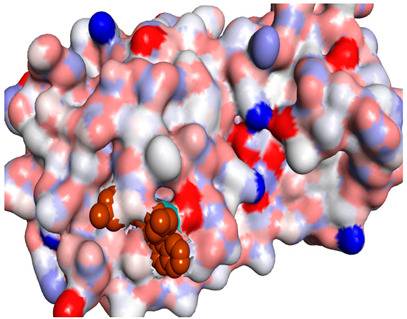
**4j**	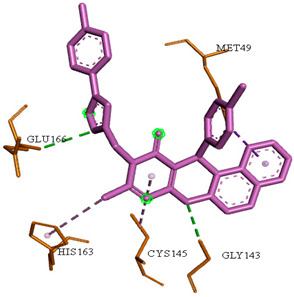	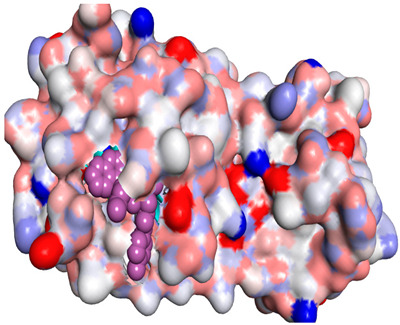

## Data Availability

Not applicable.

## Supplementary Materials

The following are available online, NMR spectra of the synthesized compounds **4a**–**l**.

## Sample Availability

Samples of the compounds **4a–l** are available from the authors.
