# Supplementary material for: Design, Microwave-Assisted Synthesis and In Silico Prediction Study of Novel Isoxazole Linked Pyranopyrimidinone Conjugates as New Targets for Searching Potential Anti-SARS-CoV-2 Agents
Source: Molecules. 2021 Oct 10;26(20):6103. doi: 10.3390/molecules26206103 (PMC8537412; doi:10.3390/molecules26206103)

Design, Microwave-Assisted Synthesis and In Silico Prediction Study of Novel Isoxazole Linked PyranopyrimidinoneConjugates as New Targets for Searching Potential Anti-SARS-CoV-2Agents

Faisal K. AlgethamI [1,*], Maher Cherif [2], Salma JlizI [2], Naoufel Ben HamadI [1,2], Anis Romdhane [2], Mohamed R. Elamin [1], Mashael A. Alghamdi [1] and Hichem Ben Jannet [2,*]

[1] Department of Chemistry, College of Science, Imam Mohammad Ibn Saud Islamic University (IMSIU), Riyadh 11432, Saudi Arabia; bh_naoufel@yahoo.fr (N.B.H.); mohamedrahmt99@gmail.com (M.R.E.); mabalghamdi@imamu.edu.sa (M.A.A.)

[2] Laboratory of Heterocyclic Chemistry, Natural Products and Reactivity (LR11ES39), Team: Medicinal Chemistry and Natural Products, Faculty of Science of Monastir, University of Monastir, Avenue of Environment, 5019 Monastir, Tunisia;cherifmhr13@gmail.com (M.C.); salma.jlizi@gmail.com (S.J.); anis_romdhane@yahoo.fr (A.R.)

* Correspondence: falgethami@imamu.edu.sa (F.K.A.); hichem.bjannet@gmail.com (H.B.J.)

^{1}H and ^{13}C Spectra of compounds 4a-l

4a : 3-methyl-6-phenyl-4-((3”-phenylisoxazol-5”-yl)methyl)-10*H*-naphtho[2,1-*b*]pyrano[2,3-*d*]pyrimidin-11(12*H*)-one

^{1}H NMR spectrum (300 MHz, $CDCl_3$)

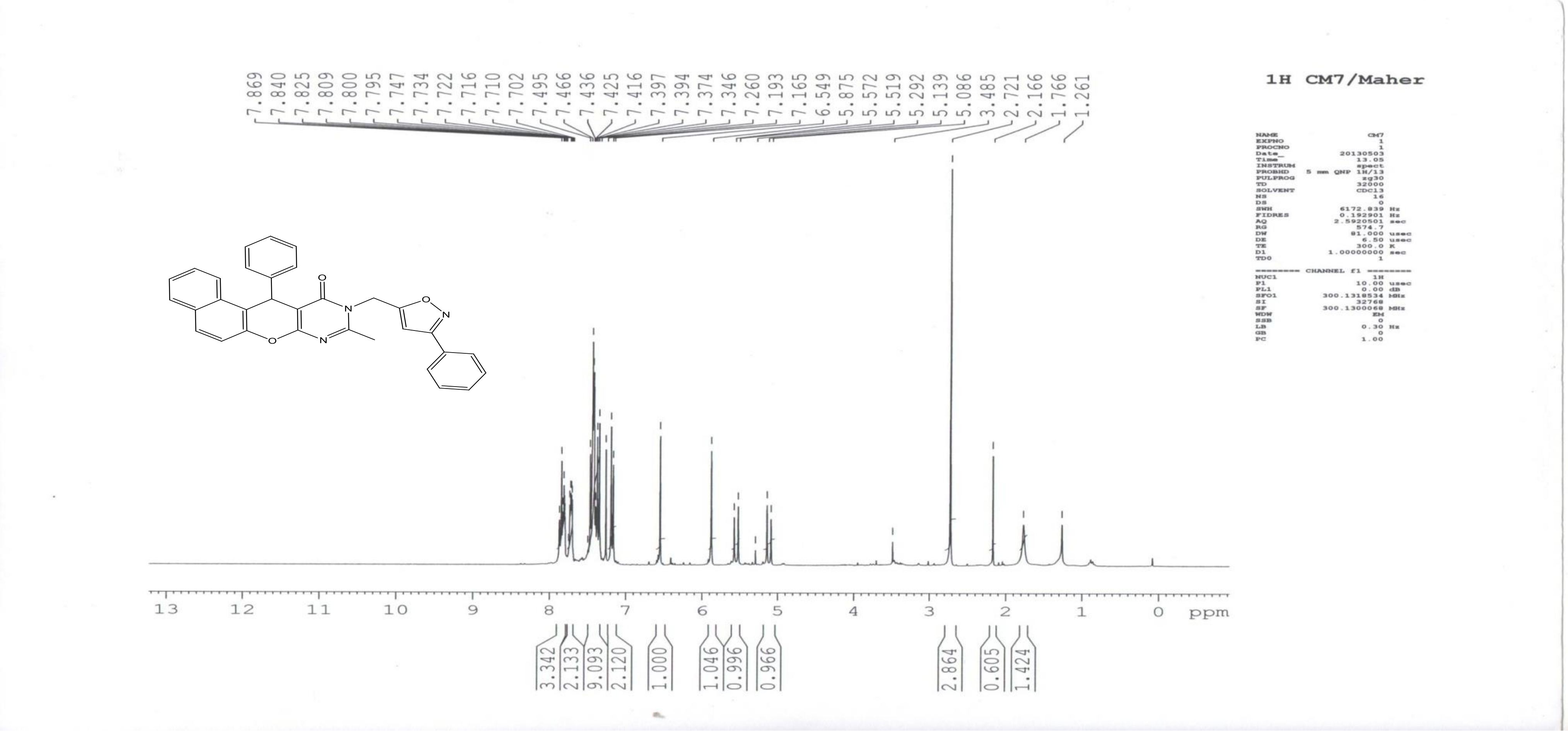

^{13}C NMR spectrum (75 MHz, $CDCl_3$)

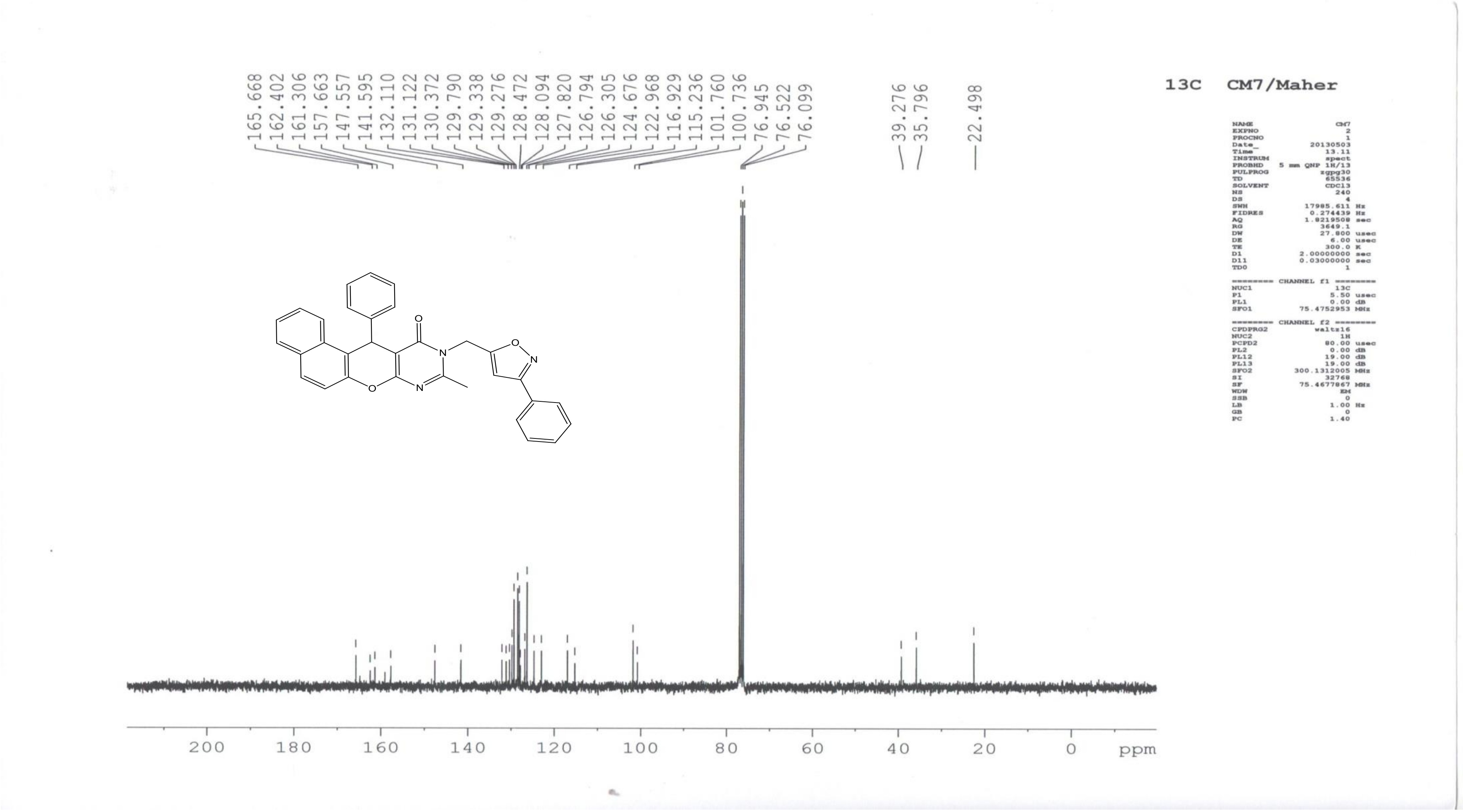

4b : 3-methyl-6-phenyl-4-((3''-(*p*-tolyl)isoxazol-5''-yl)methyl)-10*H*-naphtho[2,1-*b*]pyrano[2,3-*d*]pyrimidin-11(12*H*)-one

^{1}H NMR spectrum (300 MHz, $CDCl_3$)

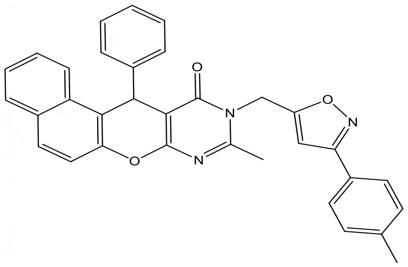

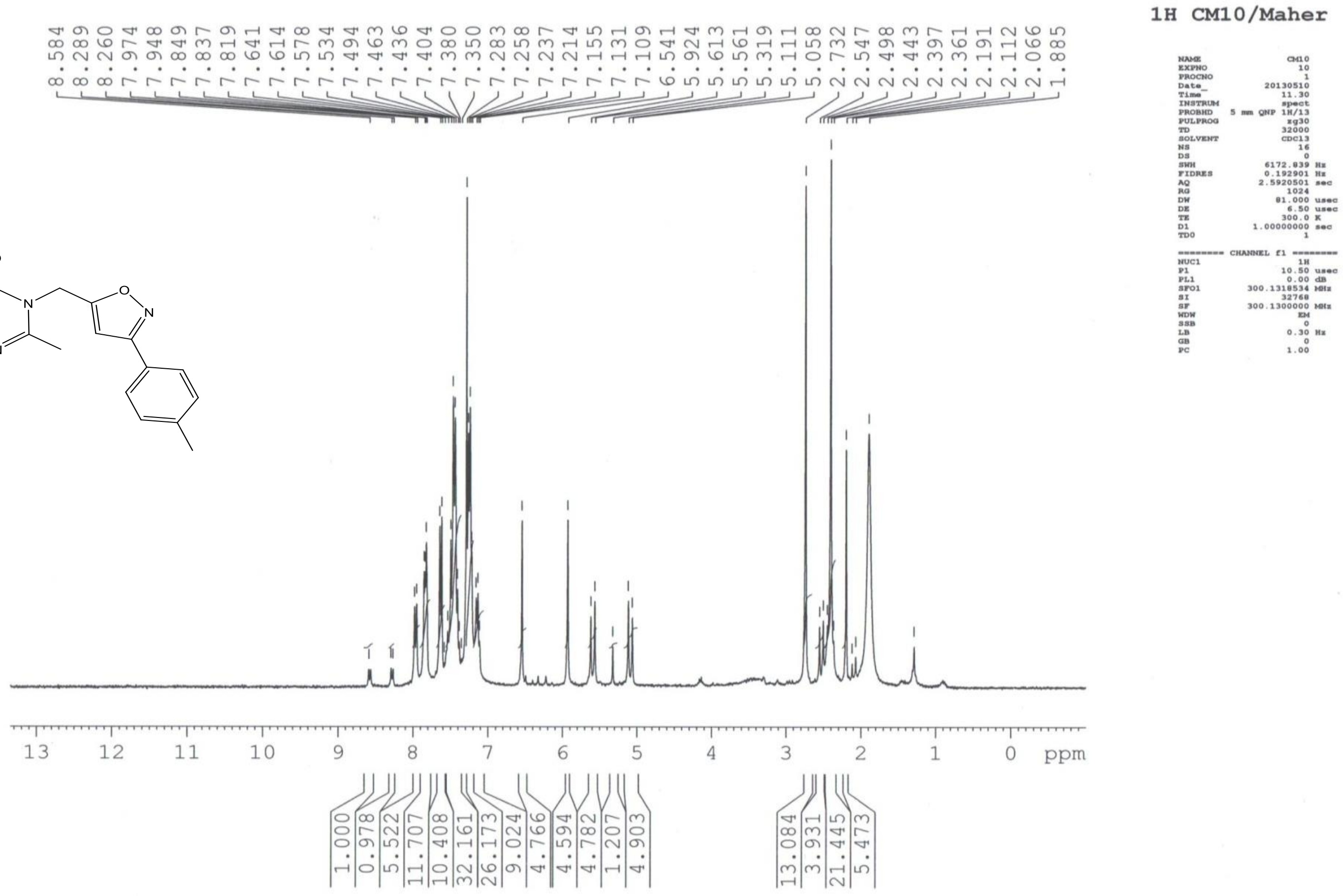

^{13}C NMR spectrum (75 MHz, $CDCl_3$)

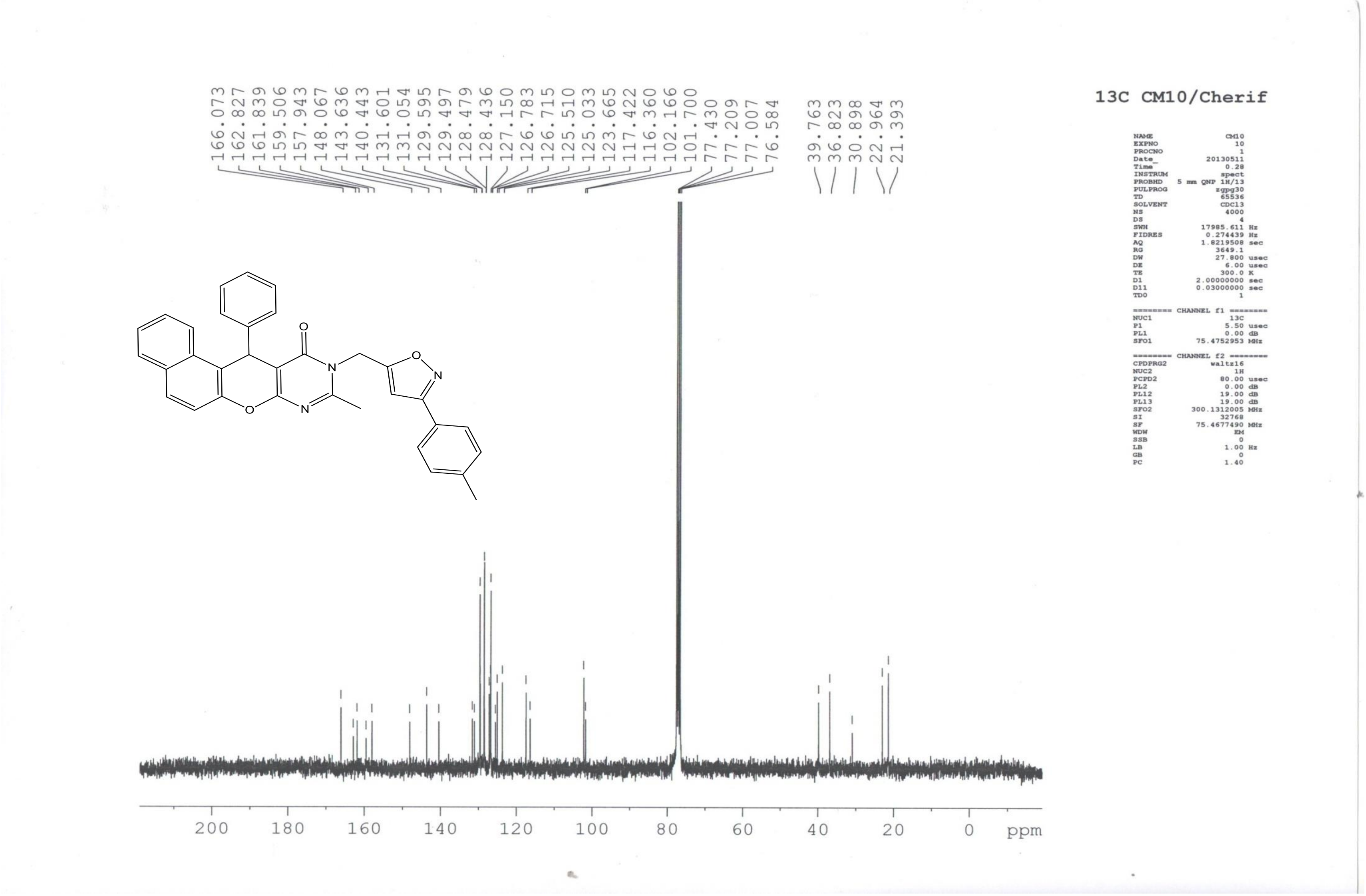

4c : 4-((3''-(*p*-methoxyphenyl)isoxazol-5''-yl)methyl)-3-methyl-6-phenyl-10*H*-naphtho[2,1-*b*]pyrano[2,3-*d*]pyrimidin-11(12*H*)-one

¹H NMR spectrum (300 MHz, $CDCl_3$)

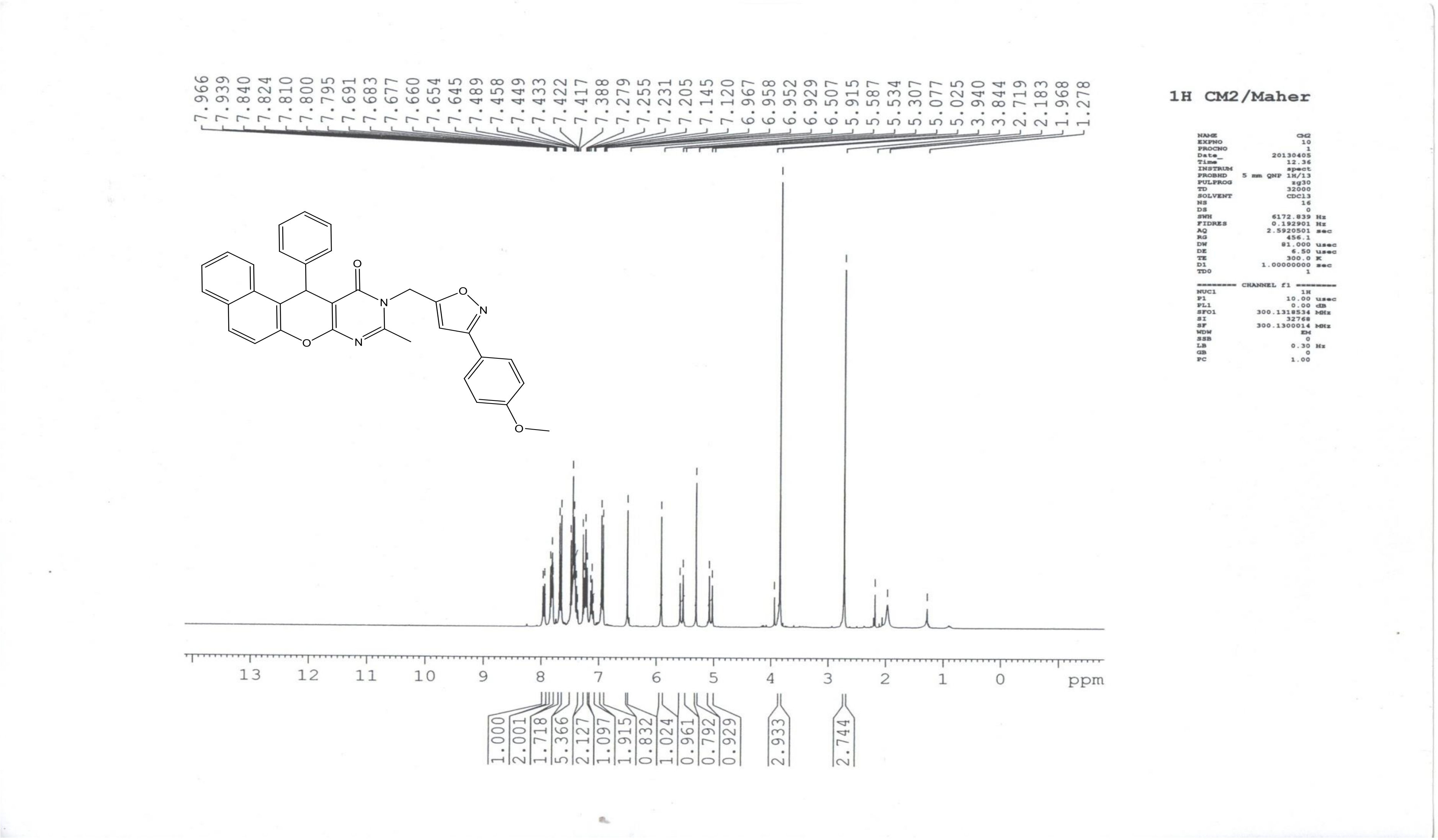

^{13}C NMR spectrum (75 MHz, $CDCl_3$)

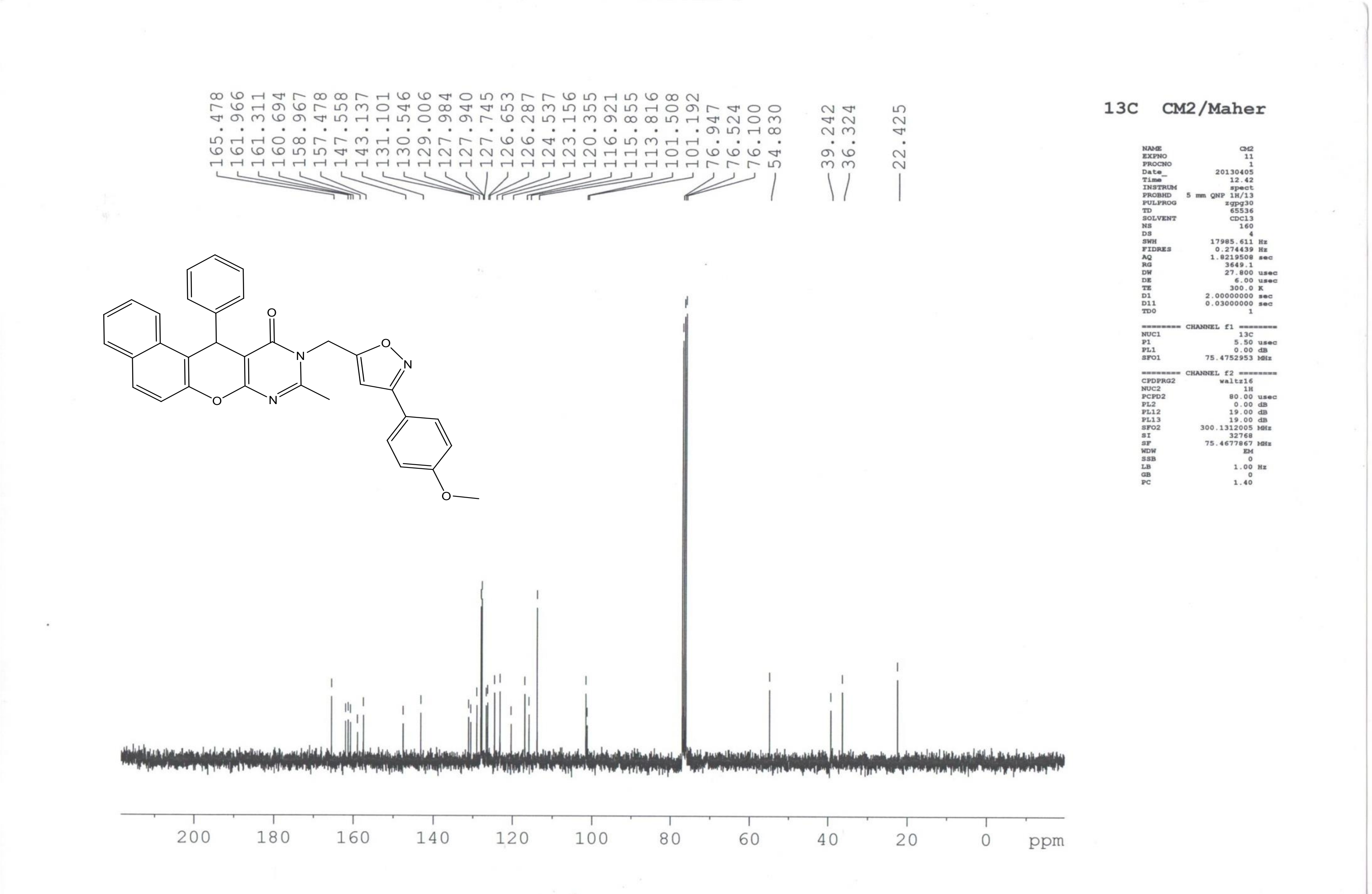

4d :4-((3”-(*p*-chlorophenyl)isoxazol-5”-yl)methyl)-3-methyl-6-phenyl-10*H*-naphtho[2,1-*b*]pyrano[2,3-*d*]pyrimidin-11(12*H*)-one

^{1}H NMR spectrum (300 MHz, $CDCl_3$)

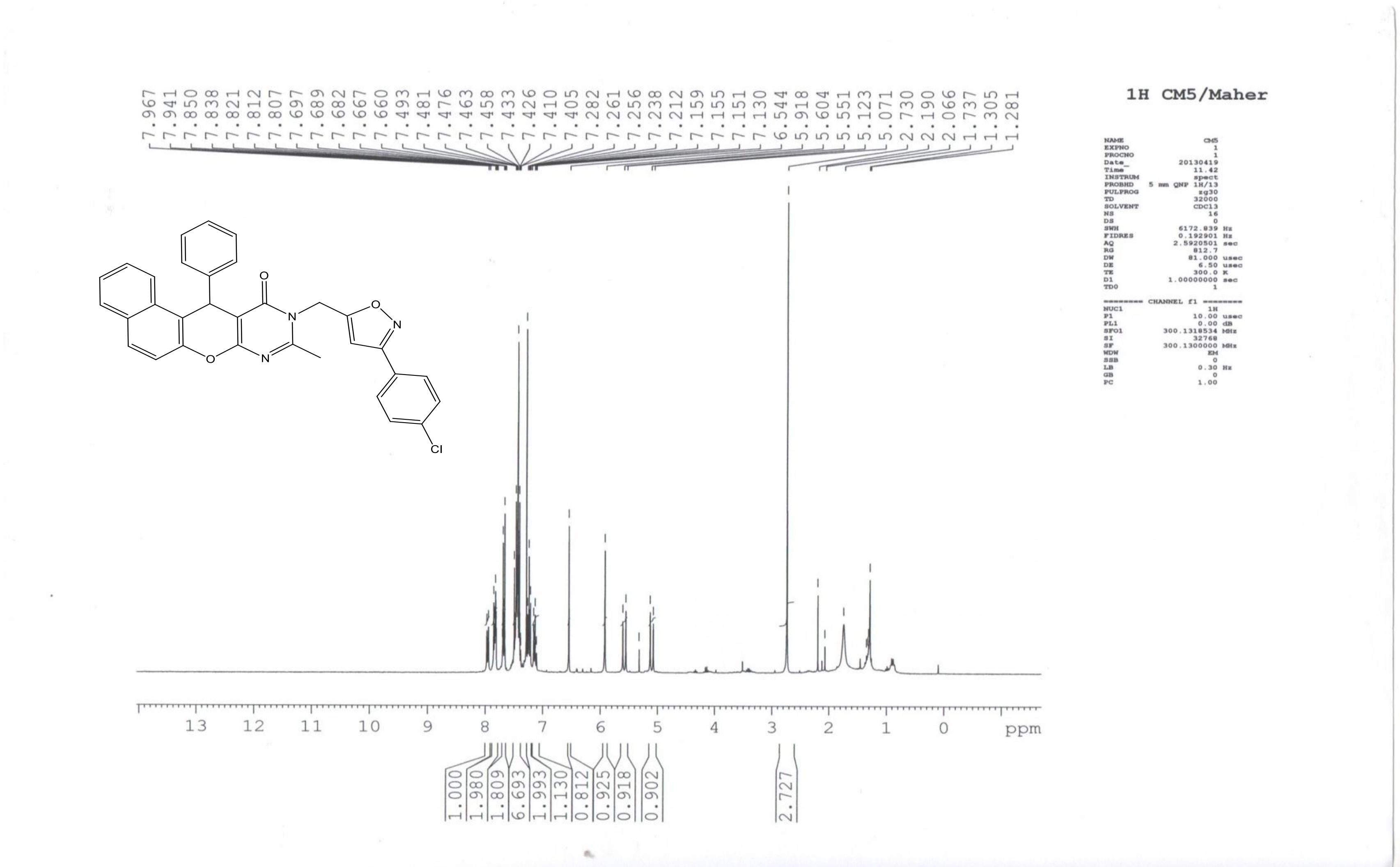

^{13}C NMR spectrum (75 MHz, $CDCl_3$)

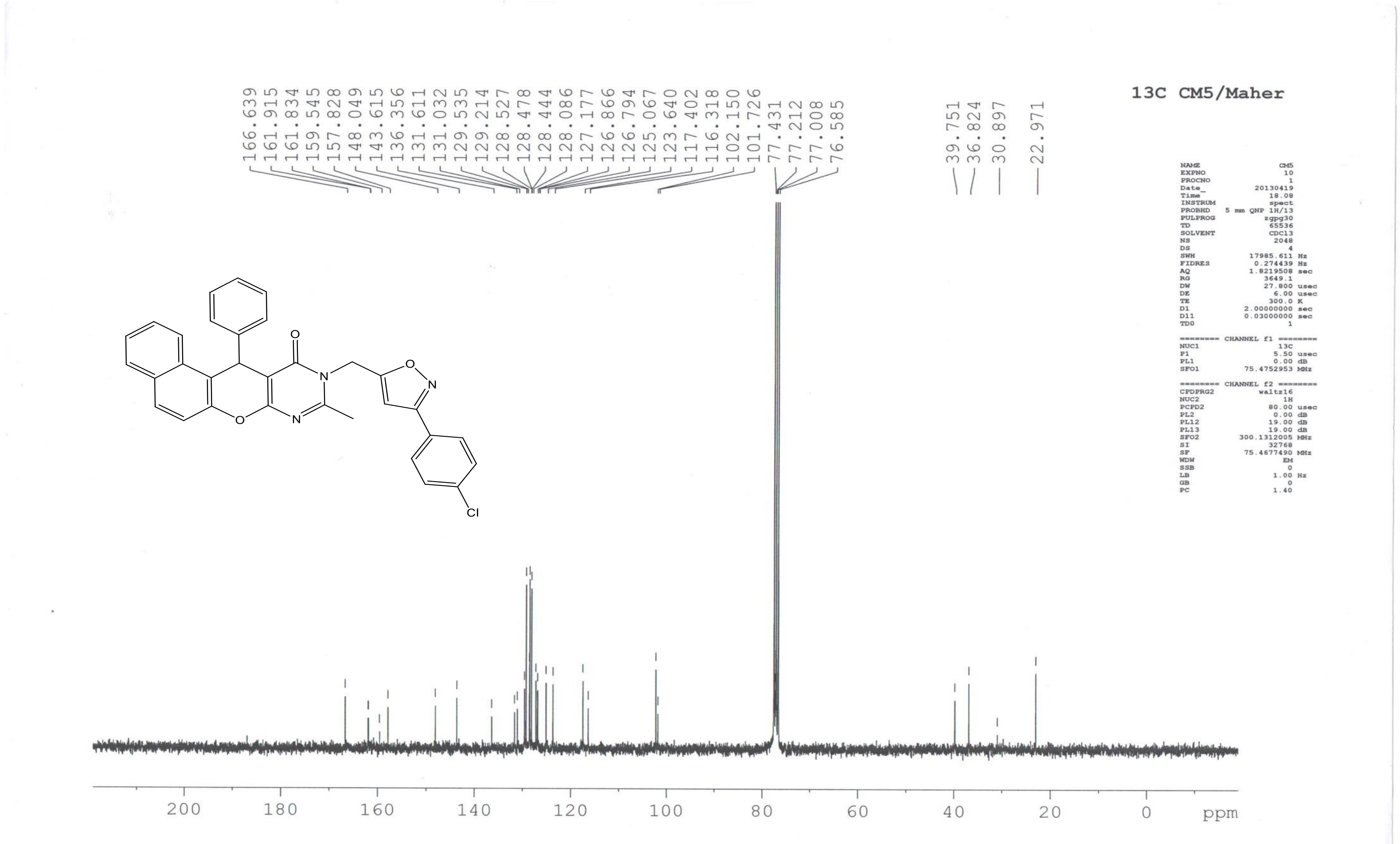

4e :3-methyl-4-((3’’-phenylisoxazol-5’’-yl)methyl)-6-(*p*-tolyl)-10*H*-naphtho[2,1-*b*]pyrano[2,3-*d*]pyrimidin-11(12*H*)-one

^{1}H NMR spectrum (300 MHz, CDCl$_3$)

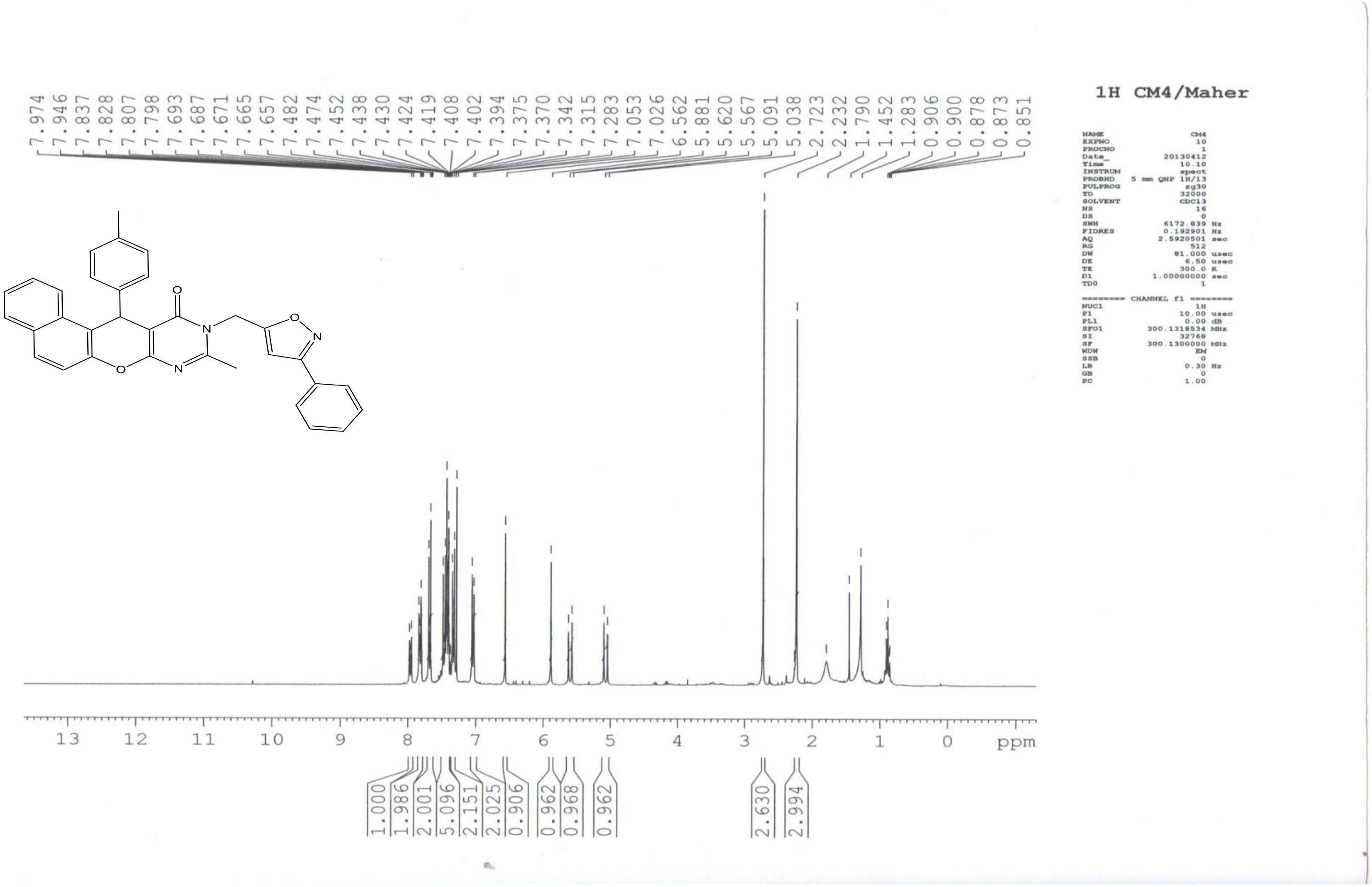

^{13}C NMR spectrum (75 MHz, $CDCl_3$)

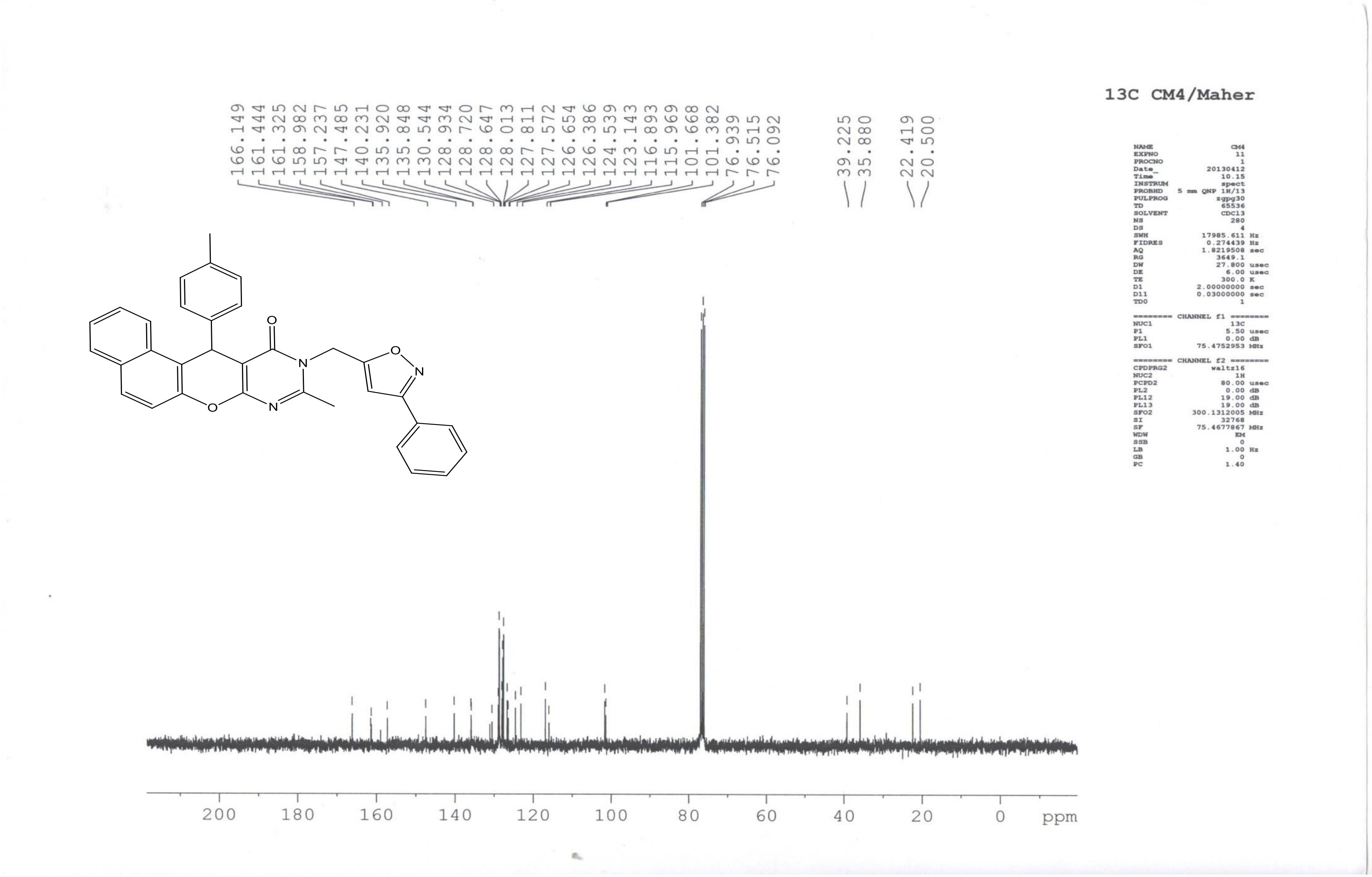

4f :3-methyl-6-(*p*-tolyl)-4-((3”-(*p*-tolyl)isoxazol-5”-yl)methyl)-10*H*-naphtho[2,1-*b*]pyrano[2,3-*d*]pyrimidin-11(12*H*)-one

^{1}H NMR spectrum (300 MHz, $CDCl_3$)

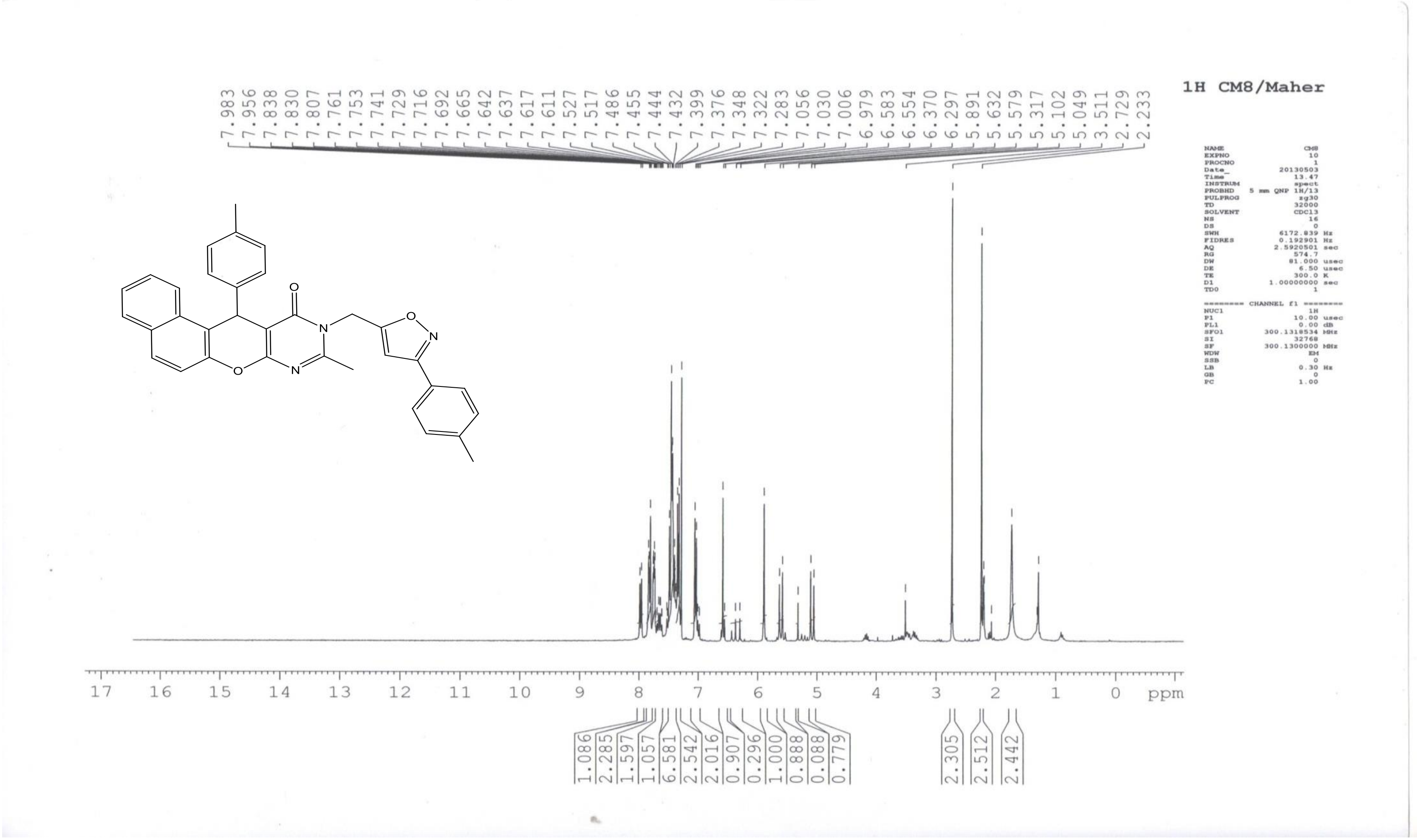

^{13}C NMR spectrum (75 MHz, $CDCl_3$)

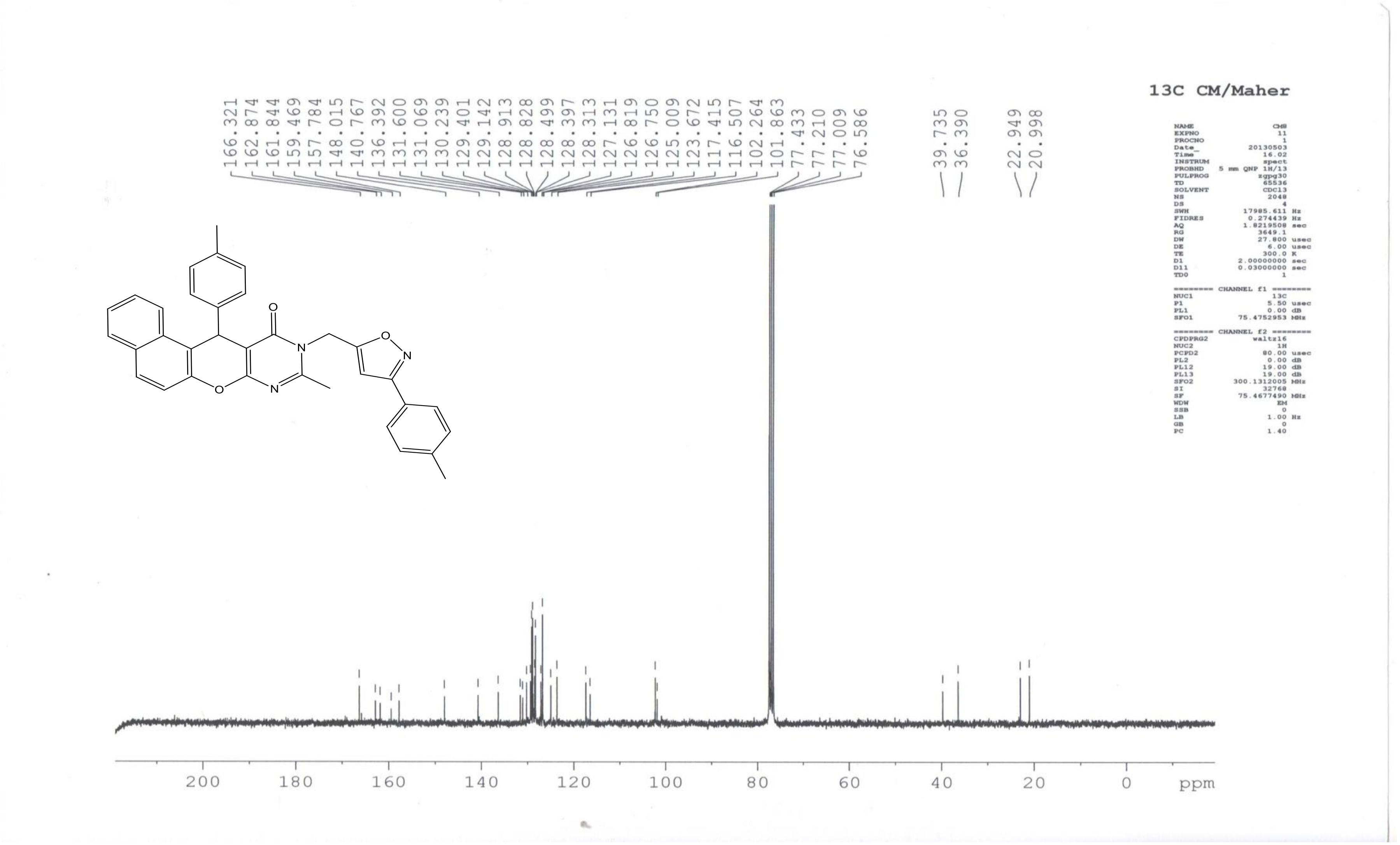

4g :4-((3”-(*p*-methoxyphenyl)isoxazol-5”-yl)methyl)-3-methyl-6-(*p*-tolyl)-10*H*-naphtho[2,1-*b*]pyrano[2,3-*d*]pyrimidin-11(12*H*)-one

^{1}H NMR spectrum (300 MHz, $CDCl_3$)

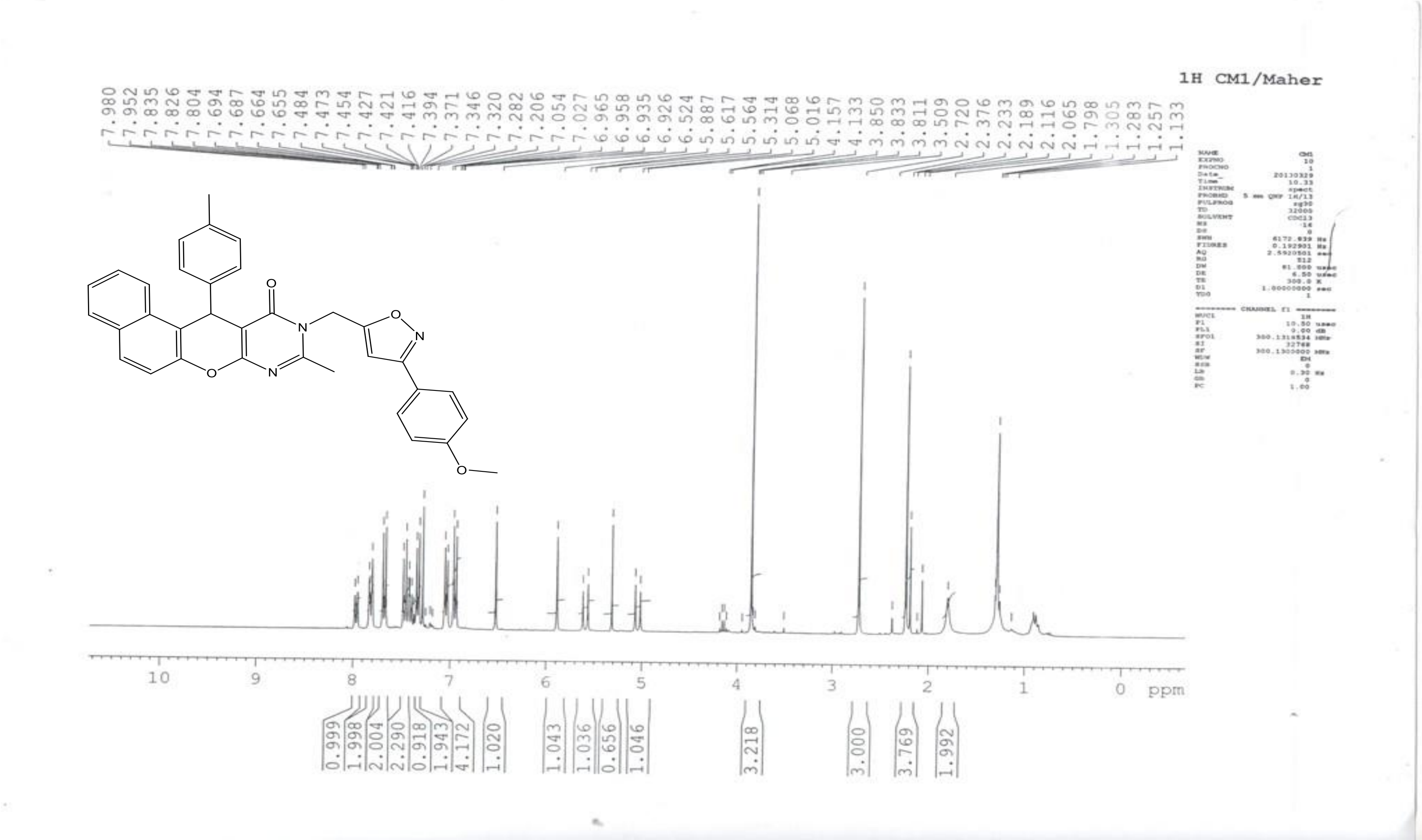

^{13}C NMR spectrum (75 MHz, $CDCl_3$)

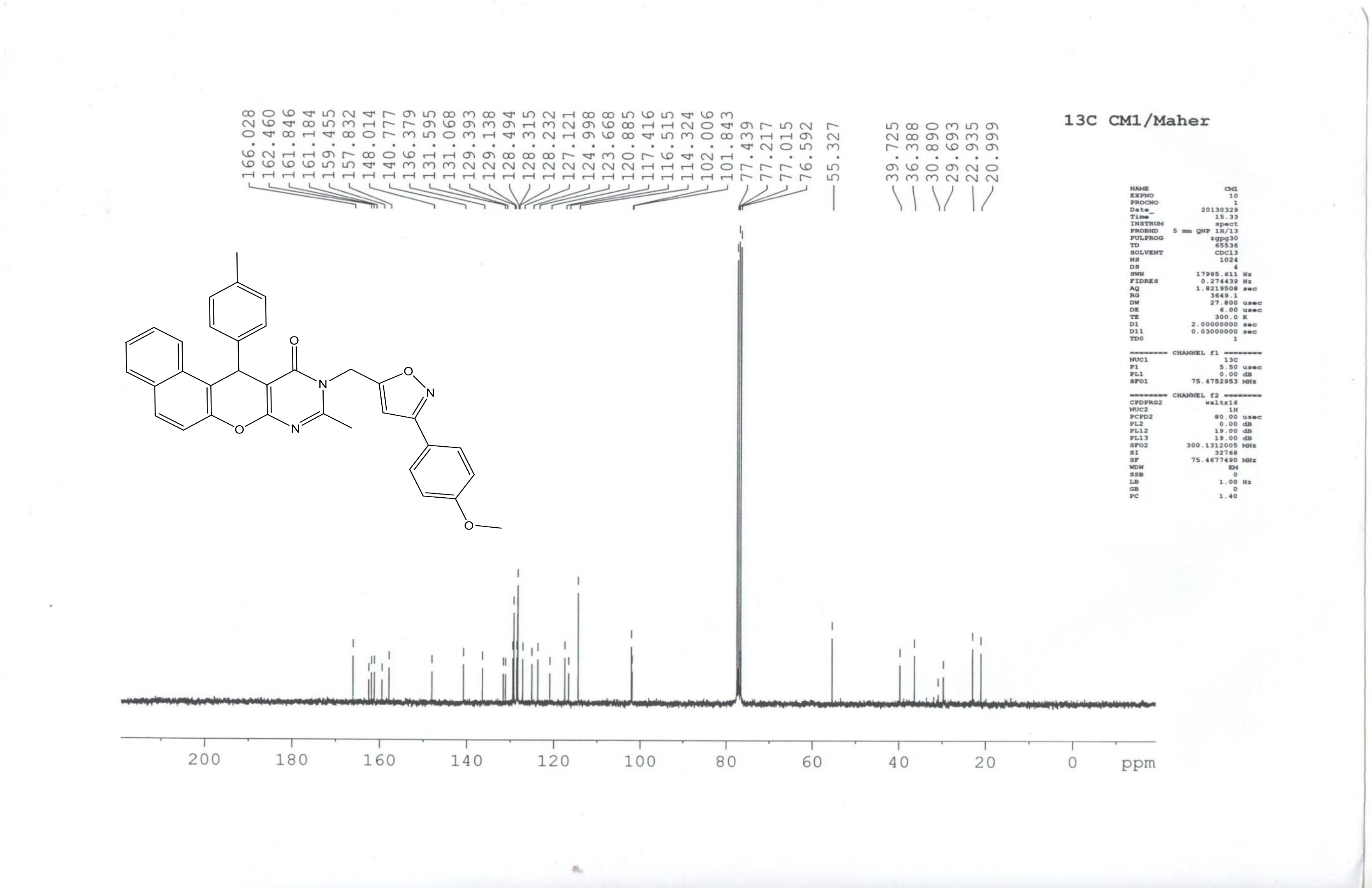

4h :4-((3-(4-chlorophenyl)isoxazol-5-yl)methyl)-3-methyl-6-(*p*-tolyl)-10*H*-naphtho[2,1-*b*]pyrano[2,3-*d*]pyrimidin-11(12*H*)-one

^{1}H NMR spectrum (300 MHz, $CDCl_3$)

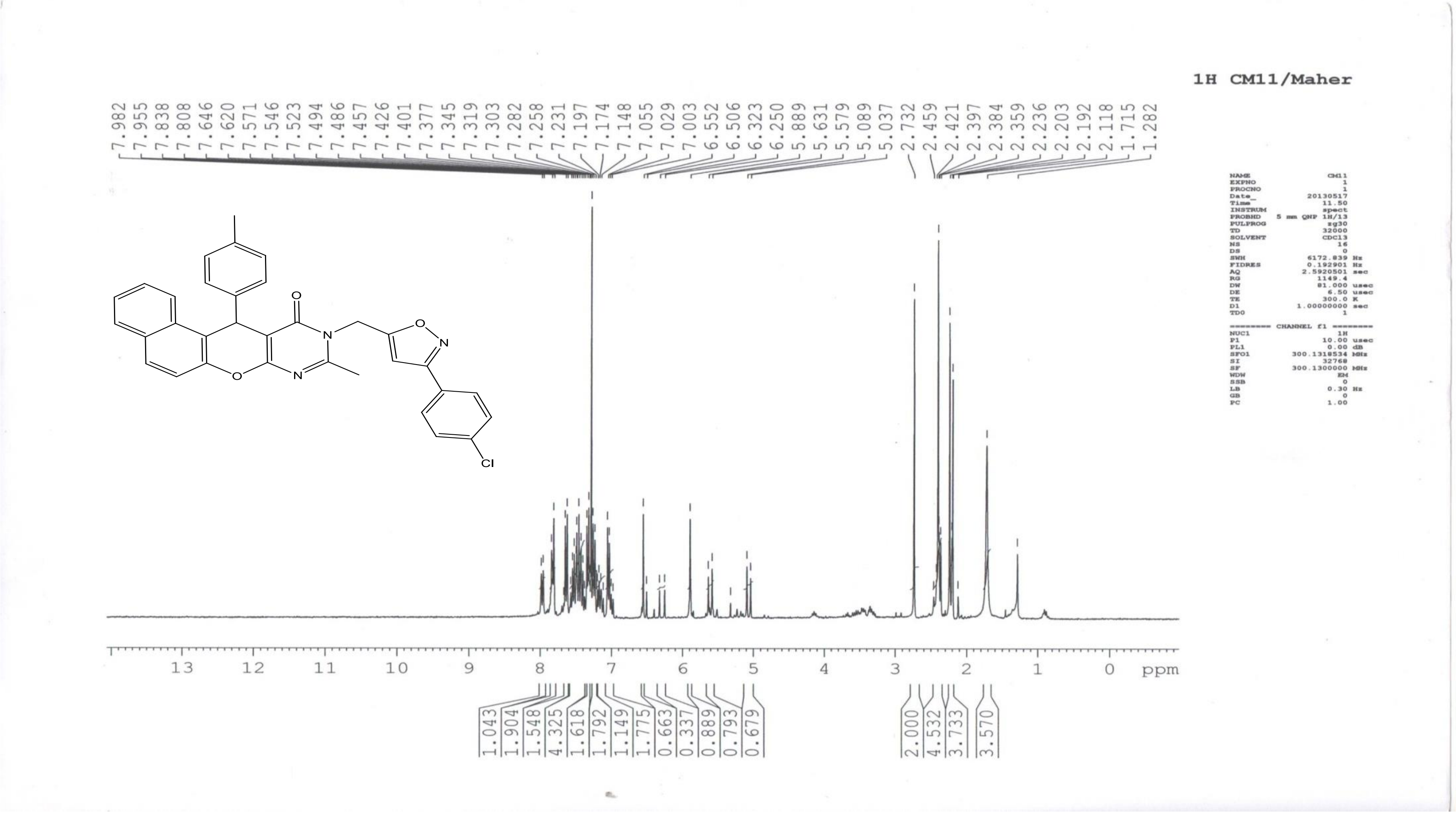

^{13}C NMR spectrum (75 MHz, $CDCl_3$)

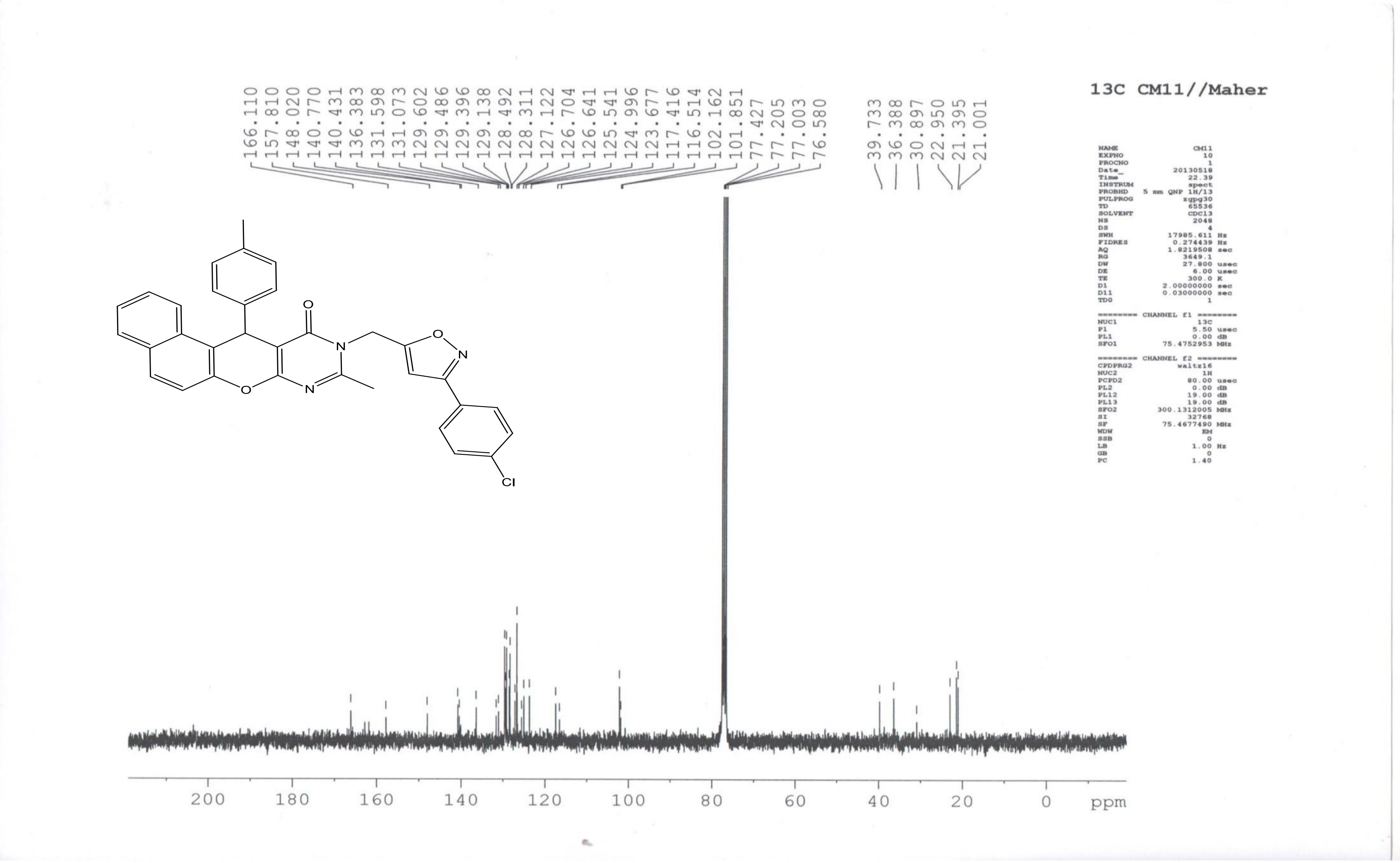

4i :6-(4-chlorophenyl)-3-methyl-4-((3”-phenylisoxazol-5”-yl)methyl)-10*H*-naphtho[2,1-*b*]pyrano[2,3-*d*]pyrimidin-11(12*H*)-one

^{1}H NMR spectrum (300 MHz, $CDCl_3$)

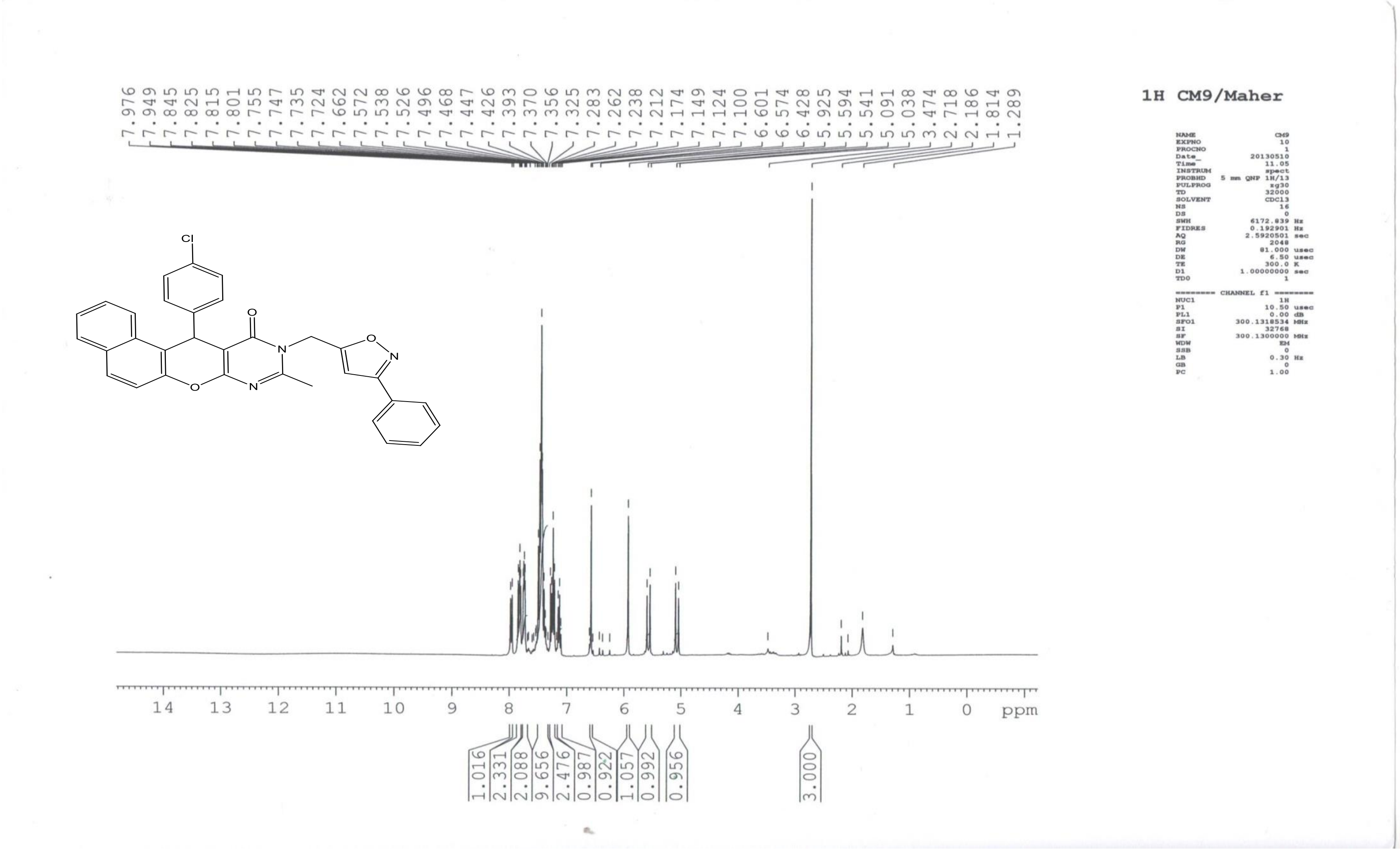

^{13}C NMR spectrum (75 MHz, $CDCl_3$)

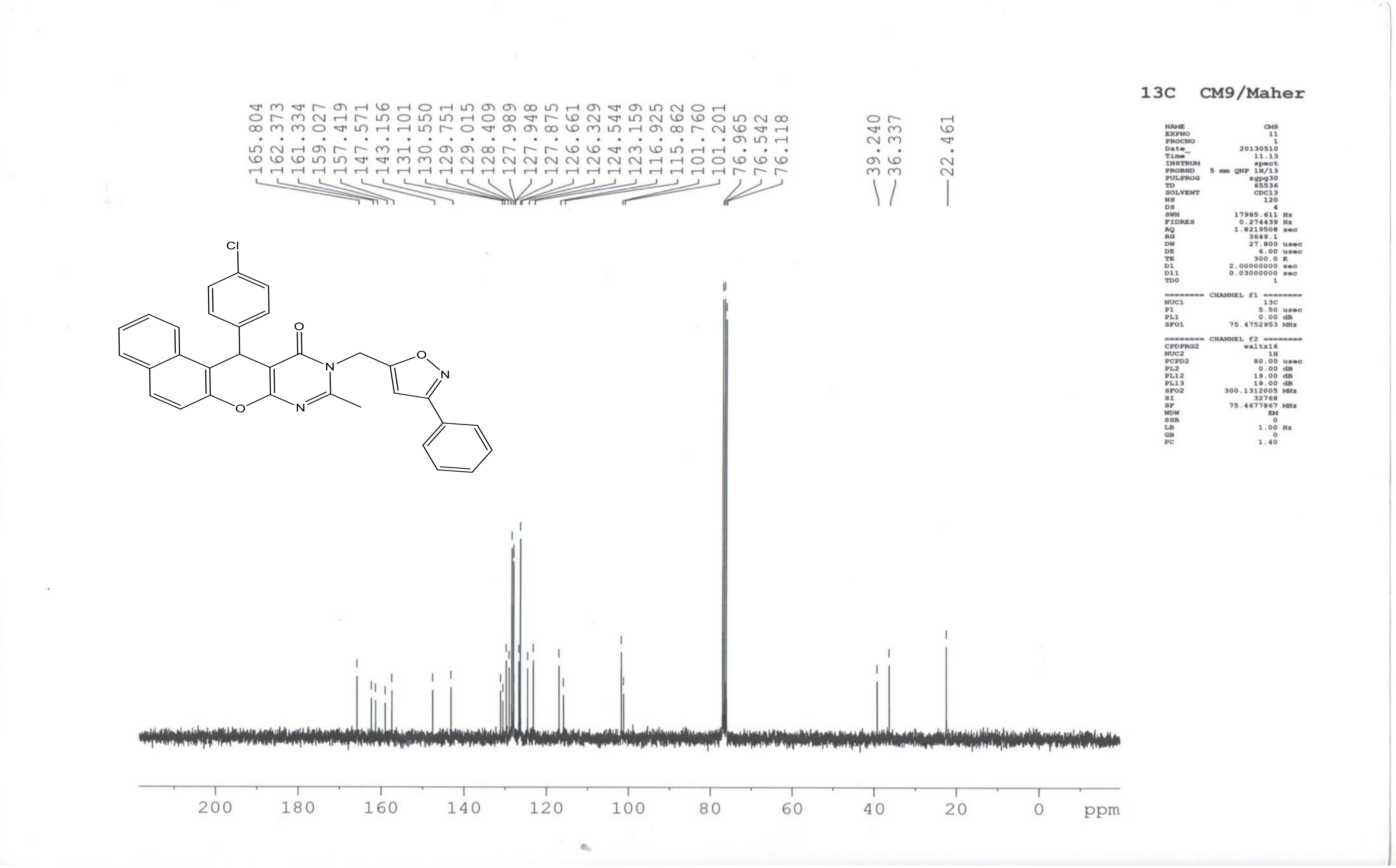

4j : 6-(*p*-chlorophenyl)-3-methyl-4-((3”-(*p*-tolyl)isoxazol-5”-yl)methyl)-10*H*-naphtho[2,1-*b*]pyrano[2,3-*d*]pyrimidin-11(12*H*)-one

^{1}H NMR spectrum (300 MHz, CDCl₃)

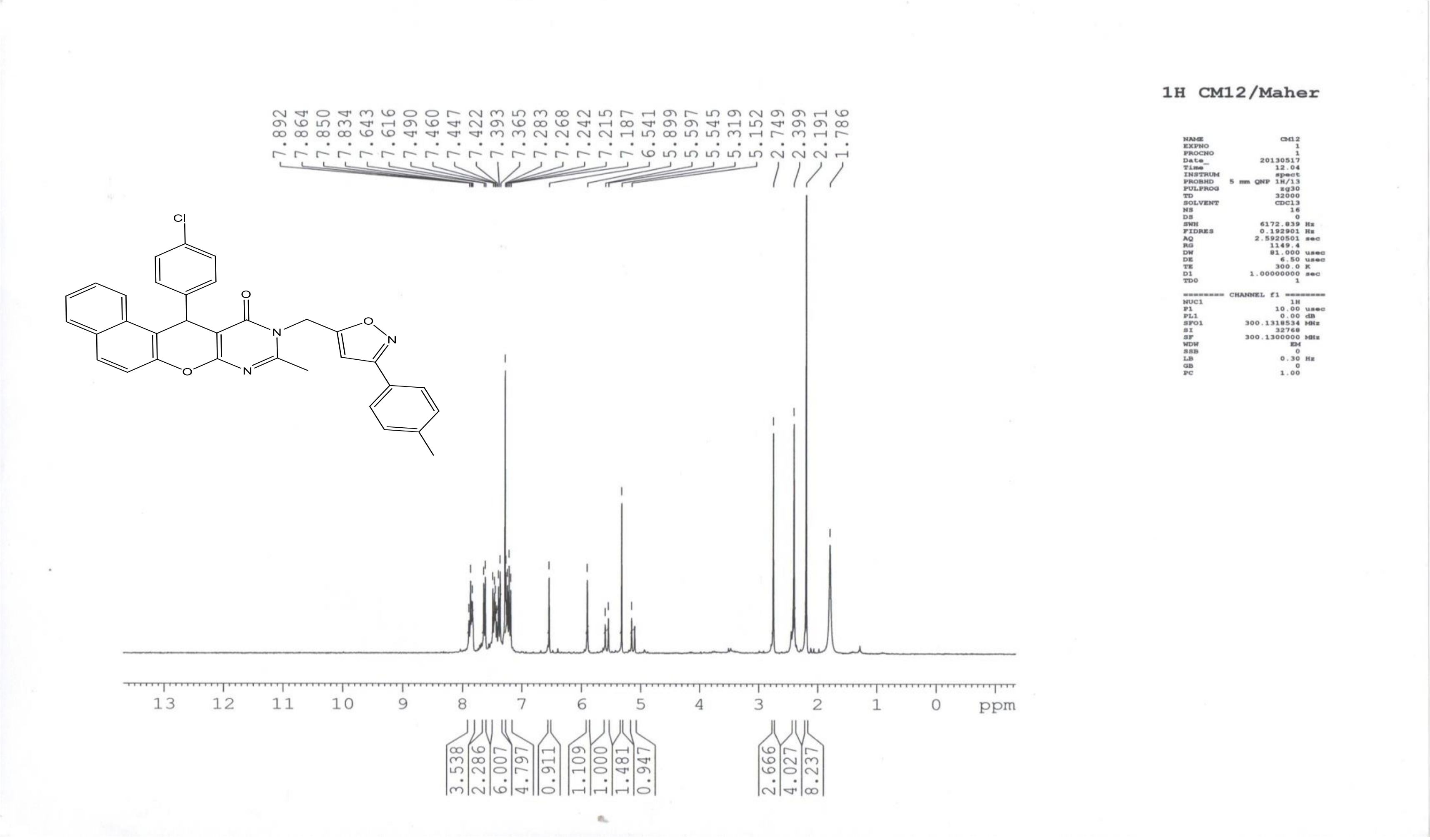

^{13}C NMR spectrum (75 MHz, $CDCl_3$)

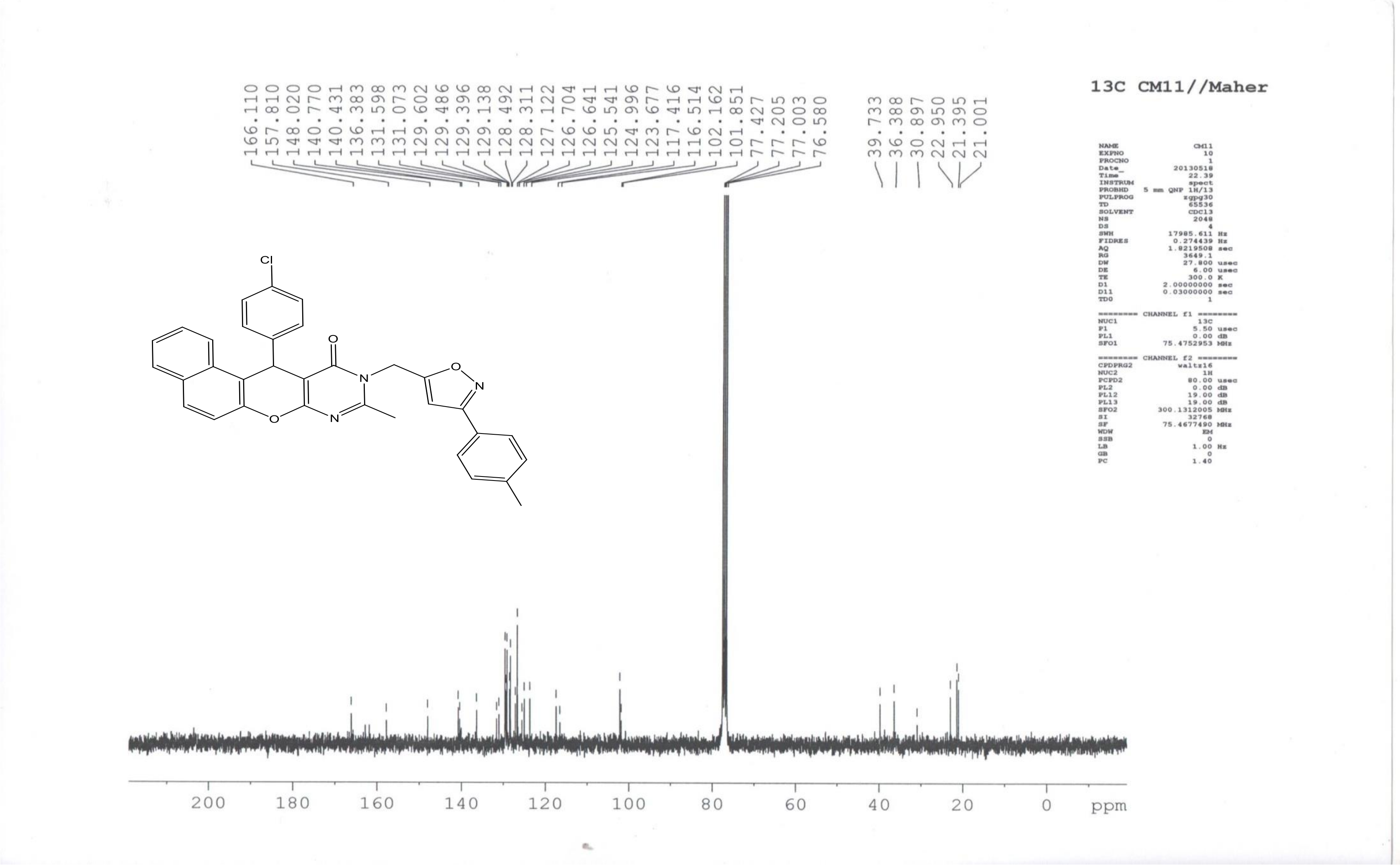

4k :6-(*p*-chlorophenyl)-4-((3”-(*p*-methoxyphenyl)isoxazol-5”-yl)methyl)-3-methyl-10*H*-naphtho[2,1-*b*]pyrano[2,3-*d*]pyrimidin-11(12*H*)-one

^{1}H NMR spectrum (300 MHz, $CDCl_3$)

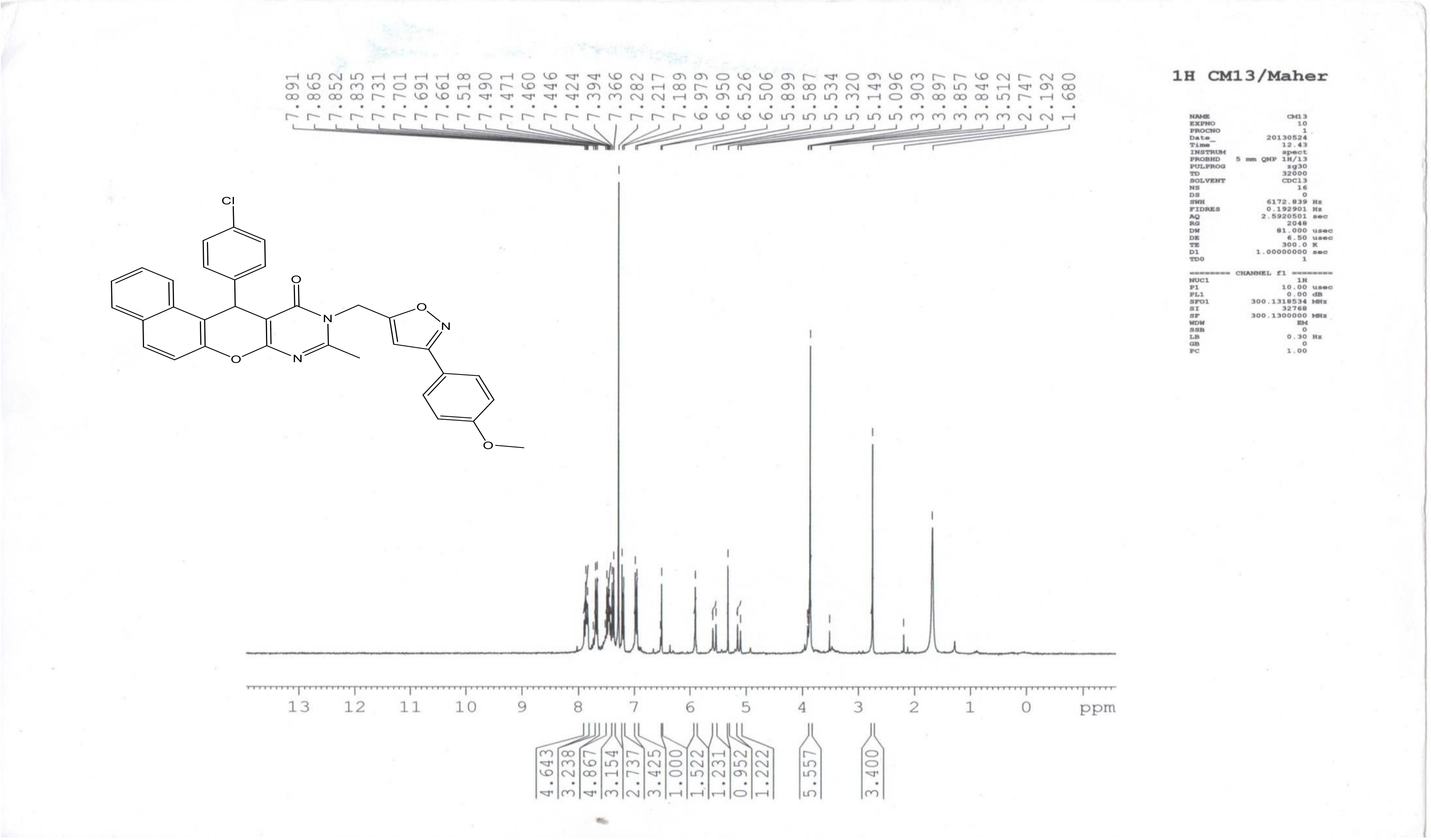

^{13}C NMR spectrum (75 MHz, $CDCl_3$)

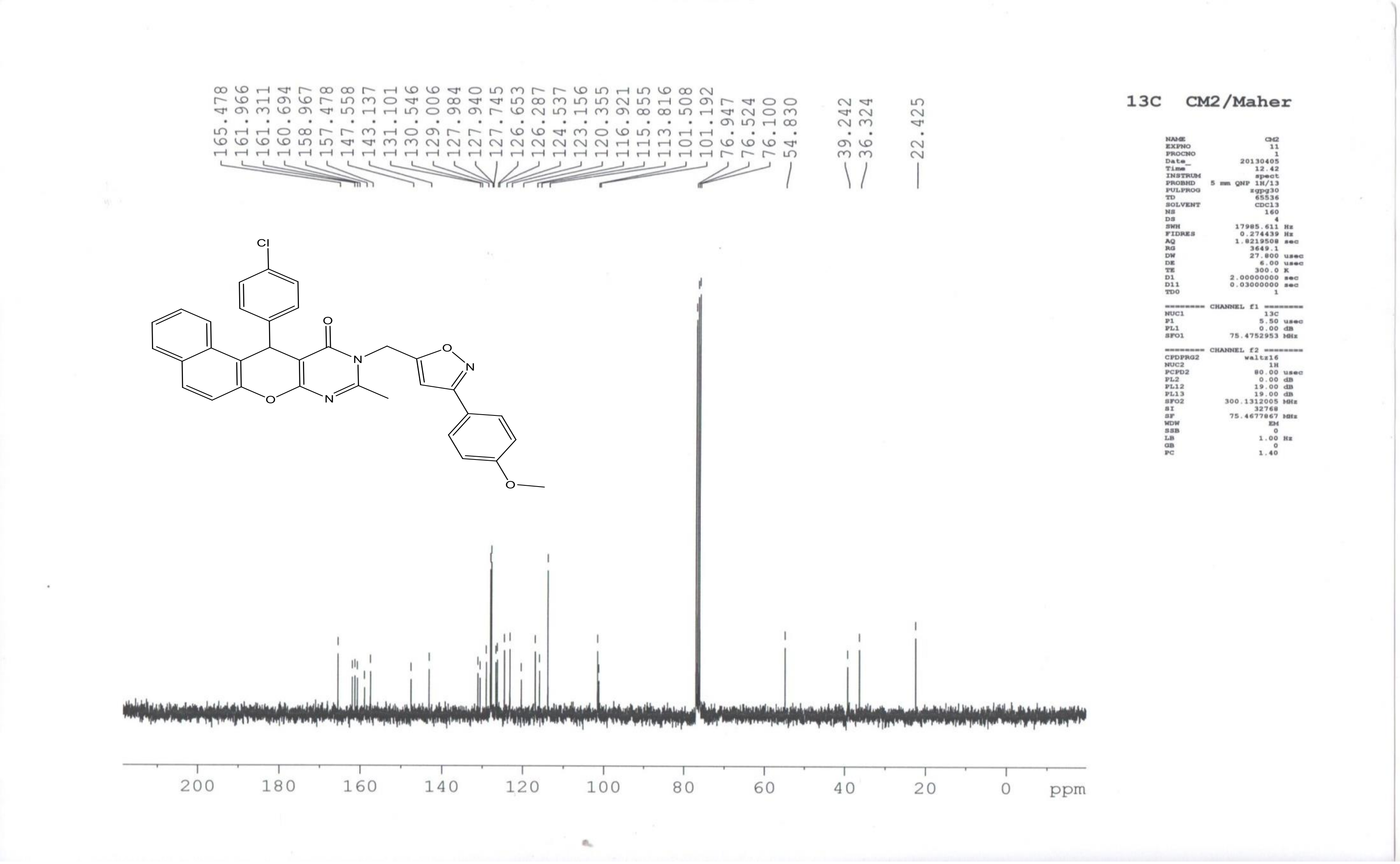

4l:6-(*p*-chlorophenyl)-4-((3”-(*p*-chlorophenyl)isoxazol-5”-yl)methyl)-3-methyl-10*H*-naphtho[2,1-*b*]pyrano[2,3-*d*]pyrimidin-11(12*H*)-one

^{1}H NMR spectrum (300 MHz, CDCl$_3$)

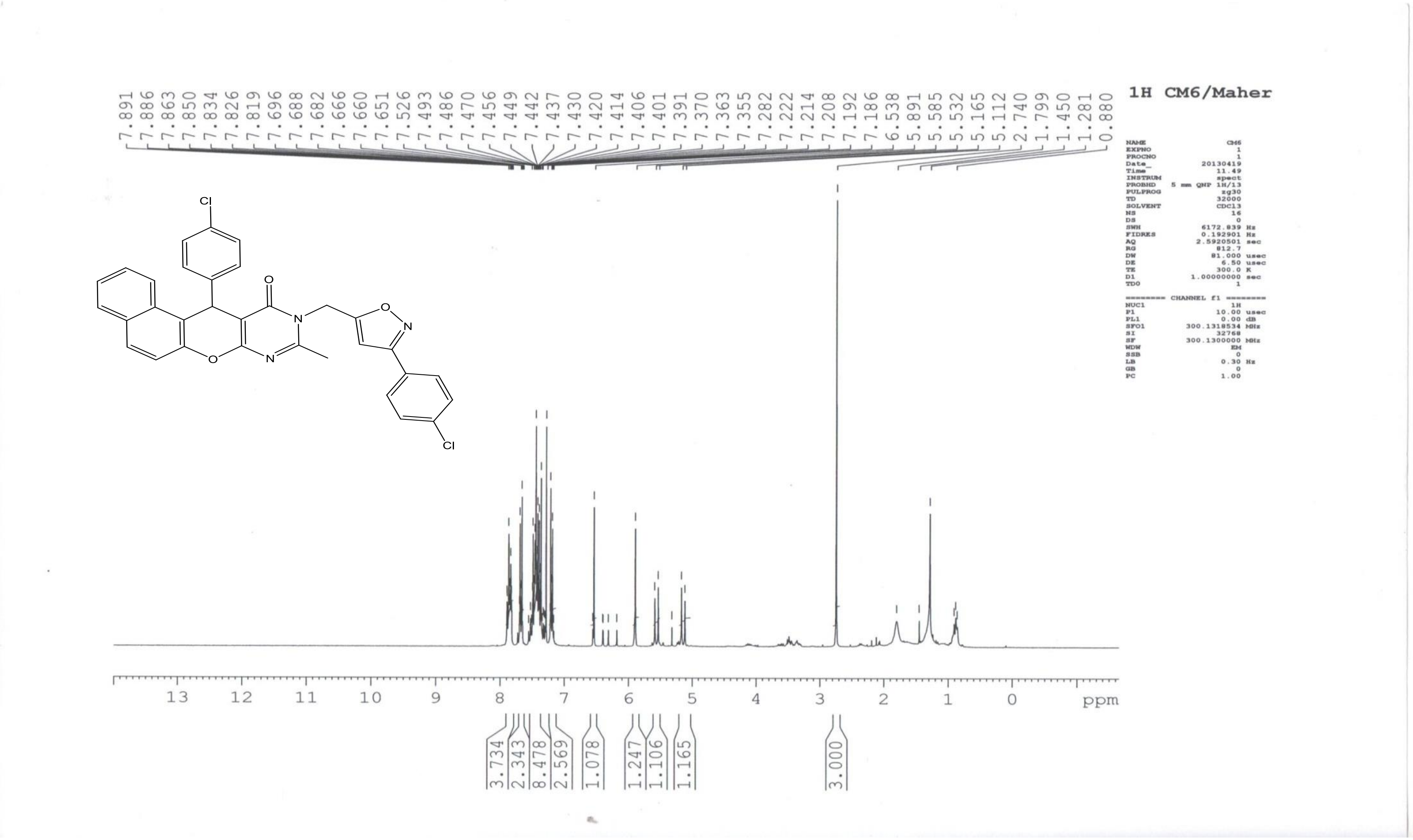

^{13}C NMR spectrum (75 MHz, $CDCl_3$)

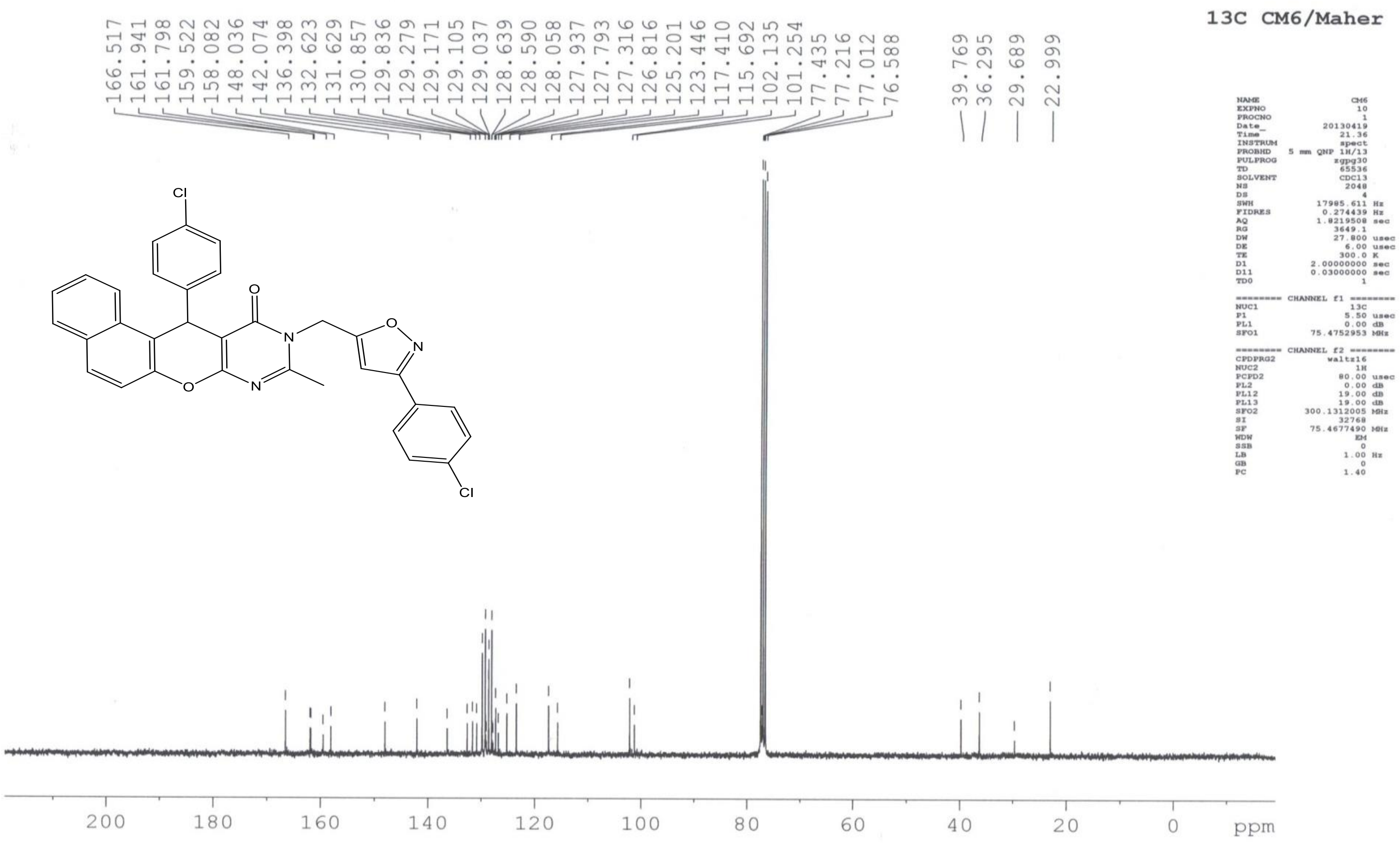

Supplement: Supplementary file 1 [file molecules-26-06103-s001.zip › molecules-1391176-supplementary.pdf]
